# Synthetic Zippers as an Enabling Tool for Engineering of Non‐Ribosomal Peptide Synthetases**

**DOI:** 10.1002/anie.202102859

**Published:** 2021-06-27

**Authors:** Kenan A. J. Bozhueyuek, Jonas Watzel, Nadya Abbood, Helge B. Bode

**Affiliations:** ^1^ Molecular Biotechnology Institute of Molecular Biosciences Goethe University Frankfurt 60438 Frankfurt am Main Germany; ^2^ Max-Planck-Institute for Terrestrial Microbiology Department of Natural Products in Organismic Interactions 35043 Marburg Germany; ^3^ Senckenberg Gesellschaft für Naturforschung 60325 Frankfurt am Main Germany

**Keywords:** artificial docking domains, biosynthesis, engineering, non-ribosomal peptide syntheses, novel natural products

## Abstract

Non‐ribosomal peptide synthetases (NRPSs) are the origin of a wide range of natural products, including many clinically used drugs. Efficient engineering of these often giant biosynthetic machineries to produce novel non‐ribosomal peptides (NRPs) is an ongoing challenge. Here we describe a cloning and co‐expression strategy to functionally combine NRPS fragments of Gram‐negative and ‐positive origin, synthesising novel peptides at titres up to 220 mg L^−1^. Extending from the recently introduced definition of e*X*change *U*nits (XUs), we inserted synthetic zippers (SZs) to split single protein NRPSs into independently expressed and translated polypeptide chains. These synthetic type of NRPS (type S) enables easier access to engineering, overcomes cloning limitations, and provides a simple and rapid approach to building peptide libraries via the combination of different NRPS subunits.

## Graphical abstract

Re‐engineering of non‐ribosomal peptide synthetases (NRPSs) is challenging due to their protein size up to a few megadalton. The artificial splitting of NRPSs to produce building blocks with moderate size is achieved by the introduction of synthetic zippers, which can be recombined in a rapid and simple plug and play manner to form an *in trans* regulated synthetic type of NRPSs.

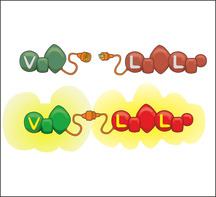

## Introduction

Non‐ribosomal peptide synthetases (NRPSs) are multifunctional enzymes, producing a broad range of structural complex and valuable compounds with applications in medicine and agriculture^[1]^ making them key targets for bioengineering. The structural diversity of non‐ribosomal peptides (NRPs) arises from the assembly line architecture of their biosynthesis. According to their biosynthetic logic, known NRPS systems are classified into three groups, linear (type A), iterative (type B), and non‐linear NRPSs (type C).^[2]^ Type A NRPSs are composed of sequential catalytically active domains organised in modules, each responsible for the incorporation and modification of one specific amino acid (AA). The catalytic activity of a canonical module is based upon the orchestrated interplay of an adenylation (A) domain for AA selection and activation, a condensation (C) domain to catalyse peptide bond formation, and a thiolation (T) domain/peptidyl‐carrier protein (PCP) onto which the AAs or intermediates are covalently tethered.^[3]^ In addition, tailoring domains, including epimerization (E), methylation, and oxidation domains can be part of a module, or a heterocyclization (Cy) domain instead of a C domain can be present. Finally, most NRPS termination modules harbour a thioesterase (TE) domain, usually responsible for the release of linear, cyclic or branched cyclic peptides.^[4]^

Type A NRPSs (Figure S1) follow the collinearity rule, i.e., the number of NRPS modules corresponds directly to the number of monomers incorporated into the associated product, and the arrangement of the modules directly follows the primary sequence of the peptides.^[5]^ Whereas in *in cis* type A NRPSs all modules are arranged on a single polypeptide chain (e.g. ACV synthetase^[6]^), *in trans* assembly lines comprise a number of individual proteins (daptomycin synthetase^[7]^). Mutual protein‐protein interactions of the latter are mediated by specialized *C*‐ (donor) and *N*‐terminally (acceptor) attached ≈30 AAs long α‐helical structural elements, so called communication‐mediating (COM) or docking domains (DDs).^[8]^ DDs typically are located in between two modules and only interact with weak affinities (4–25 μM),[^9^, ^10^, ^11^, ^12^, ^13^] but are crucial to ensure biosynthesis of the desired product(s).[^8^, ^11^, ^14^, ^15^] Despite recent progress on applying DD substitutions to program new assembly lines, in most cases structural information is lacking to effectively apply DDs for general engineering purposes.[^11^, ^16^, ^17^]

Although early engineering attempts, including the exchange of DDs, the targeted modification of the A domains substrate specificity conferring AA residues, and the substitution of domains as well as whole modules, gave mixed results, several notable advances have been published recently.[^17^, ^18^] To give but one example, we comprehensively analysed structural data as well as inter‐domain linkers in NRPSs to define novel fusion sites and to provide guidelines for exchanging A‐T‐C units, denoted as e*X*change *U*nits (XUs),^[19]^ as opposed to canonical modules (C‐A‐T).^[20]^ The XU concept is based on a conserved W]‐[NATE motif within the C‐A linker region that can be leveraged as splicing point to functionally recombine type A NRPSs.^[19]^ Based on previous biochemical characterizations of C domains[^21^, ^22^] as well as our experimental observations,^[19]^ a further requirement of the XU concept is compliance with the C domain specificity rule. Two XU building blocks should only be fused if the attributed C domains acceptor‐site specificity of the upstream XU (XU_*n*_) matches the substrate specificity of the downstream A domain (XU_*n*+1_).[^19^, ^22^] Although recent studies have questioned this gatekeeping function of the C domain,[^23^, ^24^] by following these rules it was possible to covalently combine XUs from 15 different NRPSs *in cis* to reconstitute natural and generate new peptides de novo with unprecedented efficiency.^[19]^

**Figure 1:**
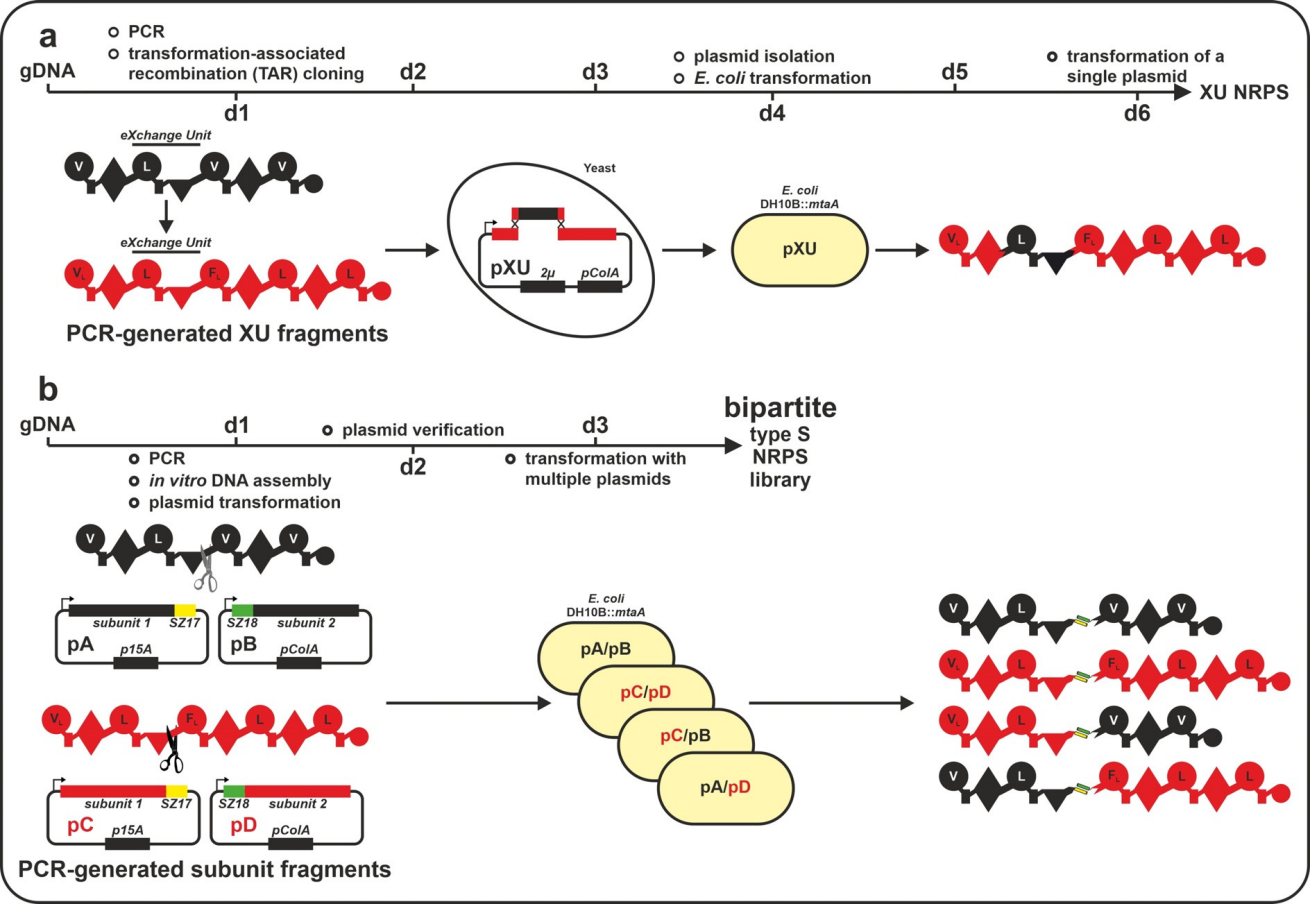
Comparison of workflows to generate chimeric NRPSs. a) “Classic” Yeast‐based TAR cloning approach following the XU concept. Ideally cloning of one recombinant BGC takes 5 days (d). Here, cloning of one BGC leads to the expression of one assembly line. b) In vitro cloning approach to generate bipartite type S NRPS takes about 3 days. Here, BGC complexity is reduced by turning them into smaller separately expressible subunits. Cloning of two bipartite type S NRPSs (four subunits) leads to four co‐expression possibilities of recombinant BGCs. For domain assignment the following symbols are used: A, adenylation domain, large circles; T, thiolation domain, rectangle; C, condensation domain, triangle; C/E, dual condensation/epimerization domain, diamond; TE, thioesterase domain, small circle.

However, cloning and engineering modular enzymatic assembly lines, due to their mere size (up to 1.81 million Da (kolossin‐producing synthetase (KolS^[25]^)) and conserved repetitive sequence stretches, naturally comes with a series of difficulties. Up to now, our engineering efforts and success in generating chimeric single protein type A NRPSs relied on transformation associated homologous recombination (TAR) based yeast cloning strategies[^19^, ^26^, ^27^] (Figure 1 a). Although TAR cloning even enabled us to generate NRP libraries by applying the recently introduced eXchange Unit Condensation domain concept,^[27]^ which applies a fusion site within the linker region of the pseudo‐dimeric C domain, compared to in vitro cloning methods (e.g. Gibson, HiFi, Hot Fusion cloning) we were facing a much longer and arduous workflow (Figure 1).

In our efforts to simplify the NRPS engineering workflow, we came across synthetic zippers (SZs).^[28]^ SZs are computationally calculated and designed synthetic peptides that interact with high affinity (*K*_D_<10 nM) via a coiled‐coil structural motif, enabling the specific association of two proteins.^[29]^ Coiled coils generally can fold into two, three or four 20–50 AA long amphipathic α‐helices and oligomerize into homo‐ or heterodimers. This structural motif is found in many proteins such as in human bZIP transcription factors.^[30]^ They are characterized by a repeating seven residue pattern, denoted as (abcdefg)_*n*_, with predominantly hydrophobic AAs at positions a and d and hydrophilic AAs at positions e and g, which contribute to tight interface formation. Previous work characterized the specificity, orientation, affinity and oligomerization state of 27 SZs, summarized in a specification sheet that is now available as a ready to use synthetic biology tool kit[^28^, ^29^] for almost unlimited combinations within linear or orthogonal networks.^[28]^

**Figure 2:**
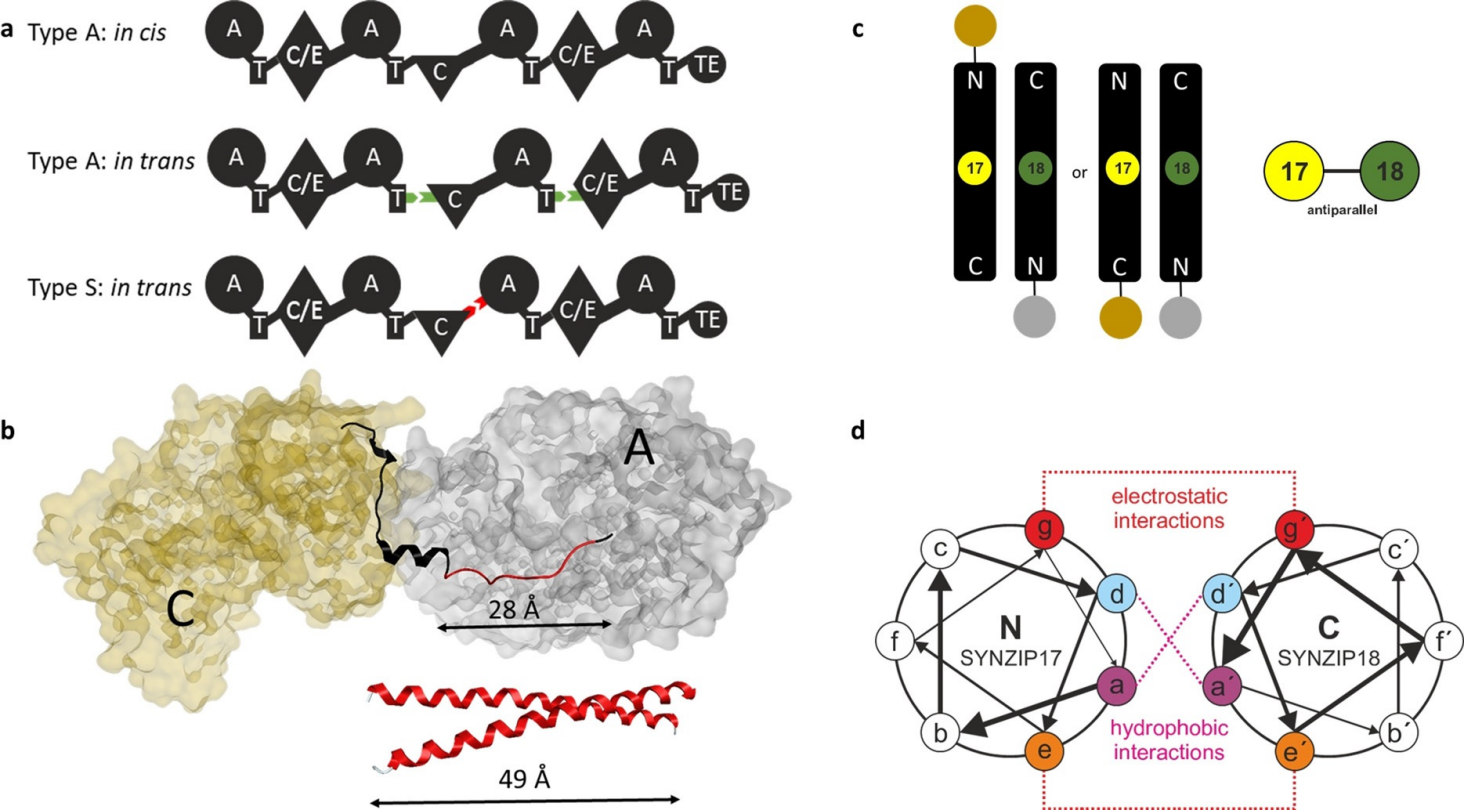
Introduction to SZ mediated in trans protein‐protein interaction. a) Overview of type A and type S NRPSs. In comparison to natural type A NRPS subunits interacting *in trans* via natural docking domains (DDs, green), the *in trans* interacting NRPS subunits in type S NRPSs are linked via synthetic zippers (SZs, red). b) Top: excised C (gold)—A (grey) di‐domain and linker region (ribbon representation) from the SrfA‐C termination module (PDB‐ID: 2VSQ). As known from the previously published e*X*change *U*nit (XU) concept the conserved W]‐[NATE motif within the C‐A linker region is an ideal intersection point to covalently recombine (*in cis*) canonical A‐T‐C subunits. To meet the native domain distance criteria by the *in trans* connection of e*X*change *U*nits via the introduction of SZs in the C‐A linker region, a 28 Å spanning stretch of amino acids (highlighted in red) has to be removed (XtpS: XU 2‐PQQPVAVIDILSSTERTLLLKTW]‐[NATETVYPES‐XU 3). Bottom: Model of 41 AAs comprising SZ17:18 pair showing its size in relation to the C‐A linker region. c) Absolute orientations of the non‐covalently connected proteins of interest (grey and brown circles) by the application of the antiparallel interacting SZ17:18 pair. The proteins of interests are either positioned on opposite or similar sites of the antiparallel SZ complex. d) Helical wheel diagram of the coiled coil formed by α‐helical SZs highlighting the characteristic interactions between hydrophobic (purple (a), blue (d)) and polar (orange (e), red (g)) amino acids in the 7‐residue (heptad, a^(^′^)^‐g^(^′^)^) repeats.

Herein, we explored the ability of SZs^[28]^ to manipulate collinear type A NRPSs by introducing artificial *in trans* regulation, as it was recently also shown for polyketide synthases.^[31]^ Such a strategy not only would allow creating a synthetic type of *in trans* regulated mega‐synthetases (type S), by combining NRPSs with high‐affinity SZs^[28]^ (Figure 2 a), but to overcome cloning and protein size limitations associated with heterologous NRP production. Eventually, this would provide a means to “reuse” already cloned protein encoding sequences by building up plasmid collections while reducing the workload—paving the way for novel high‐throughput biocombinatorial approaches at an unprecedented speed (Figure 1 b).

## Results and Discussion

Seeking to overcome present limitations of mega‐synthetase cloning and bioengineering, we explored possibilities to reduce the complexity of targeted BGCs, via functionally splitting them into separately co‐expressible subunits (Figure 1 b). Extending from the recently published XU concept^[19]^ (Figure 1), we decided to split NRPSs into two subunits between consecutive XUs at the previously defined W]‐[NATE motif of the conformationally flexible C‐A linker[^19^, ^32^, ^33^] region (Figure 1 b & Figure 2). As already known,[^19^, ^32^] this splicing position bears several advantages.[^32^, ^33^] Yet, in depth structural analysis of the crystallised termination module SrfA‐C (PDB‐ID: 2VSQ) of the surfactin biosynthesis cluster indicated that major parts of the C‐A linker must be removed to meet the distance‐criteria set out by the WT C‐A inter‐domain linker to ensure correct C‐A di‐domain contacts before SZs could be introduced (Figure 2 b). With the aim of keeping the introduced steric hindrance as minimal as possible,[^32^, ^33^] we chose the shortest readily available anti‐parallel interacting SZ pair 17 & 18^[28]^ (Figure 2 c & d), and also removed ≈10 AAs from the unstructured *N*‐terminus of resulting subunits 2, carrying the modules and domains downstream of the splicing position (Figure 2 b).[^27^, ^28^]

### Proof of Concept (I): Splitting NRPS in between XUs (A‐T‐C)

**Figure 3:**
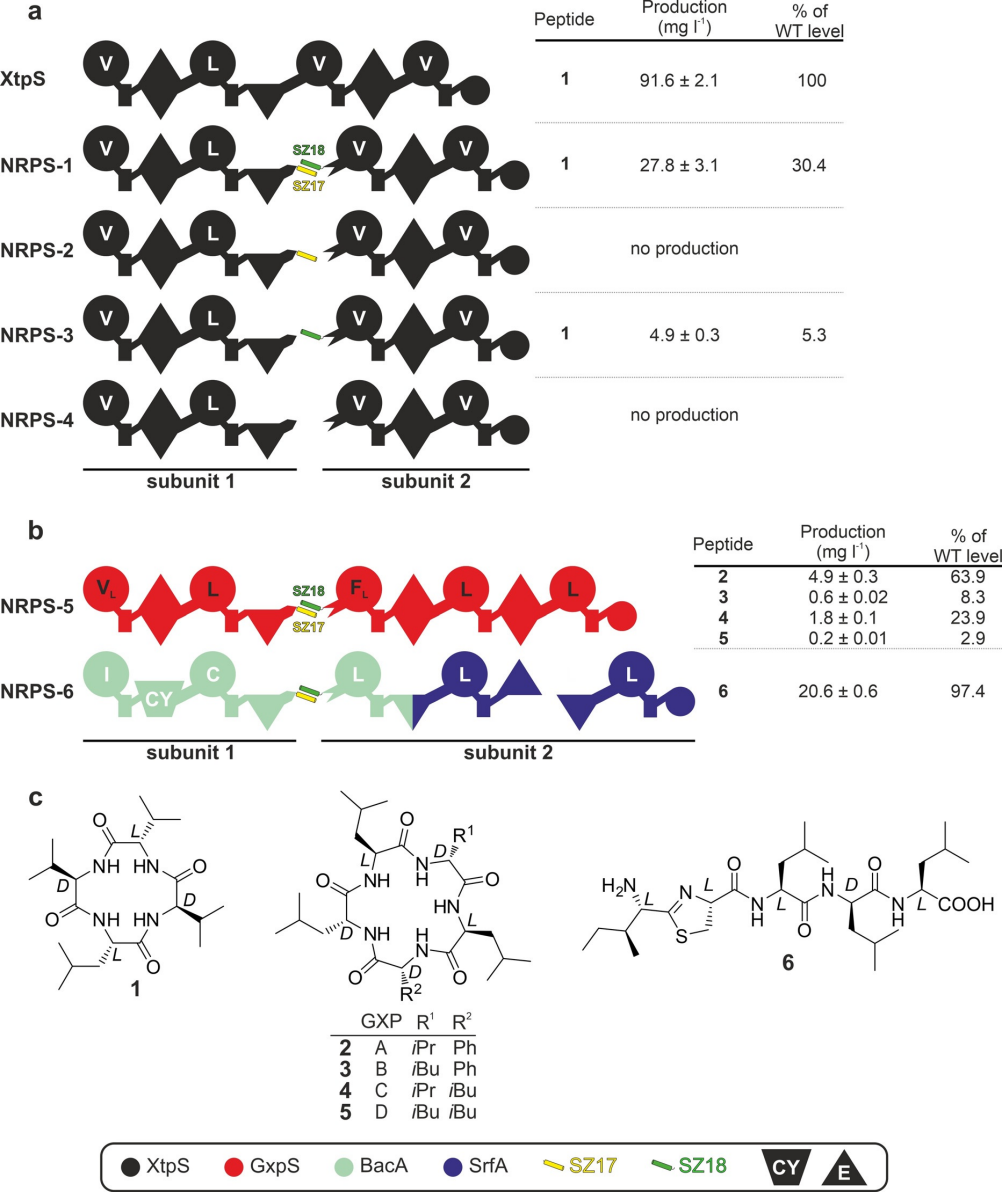
Splitting NRPS in between XUs. a) Type S **NRPS‐1** – **‐4**, as well as corresponding peptide yields obtained from triplicate experiments. b) Type S **NRPS‐5** and **‐6**, where GxpS and RtpS are split in two subunits. c) Structures of **1**–**6** produced from **NRPS‐1** to **NRPS‐6** expressed in *E. coli*. See Figure 1 for assignment of the domain symbols; further symbols: CY, heterocyclization domain; E, epimerization domain. Boxed are the colour coded NRPSs used as building blocks and the used SZ pairs.

To assess the general suitability of SZ pairs to *in trans* connect two NRPS proteins and mediate biosynthetically functional protein‐protein interface interactions, we targeted the xenotetrapeptide (**1**)‐producing NRPS (XtpS; Figure S1) from the Gram‐negative entomopathogenic bacterium *Xenorhabdus nematophila* HGB081.^[34]^ We split XtpS into two subunits in between XUs 2 and 3. Four artificial two component type S NRPSs (Figure 3 a) were constructed and heterologously produced in *E. coli* DH10B::*mtaA* ^[35]^—either with SZs fused to both subunits (NRPS‐1: subunit 1‐SZ17, SZ18‐subunit 2); only fused to subunit 1 (NRPS‐2: subunit 1‐SZ17, subunit 2) or subunit 2 (NRPS‐3: subunit 1, SZ18‐subunit 2), and without SZs (NRPS‐4: subunit 1, subunit 2).

NRPS‐2 and NRPS‐4 showed no detectable peptide production, whereas NRPS‐1 led to the production of **1** with ≈30 % (28 mg L^−1^) yield compared to WT XtpS (Figure 3 a, Figure S2), confirming that SZs indeed can be used to functionally mediate new‐to‐nature *in trans* regulation of NRP biosynthesis. NRPS‐3 with SZ18 fused to subunit 2, but lacking SZ17 on subunit 1, showed much lower yields of **1**. Despite lacking SZ17, the *C*‐terminus of XtpS subunit 1 might form a leucine‐rich α‐helical structure (*cf*. Figure 2 b & PDB‐ID: 2VSQ) that could interact with SZ18 of subunit 2 and mediate an impaired but catalytically active C‐A interface.[^19^, ^32^, ^33^, ^36^]

Additionally, SZ17:18 were used to split the GameXPeptide A–D (**2**–**5**)‐producing NRPS (GxpS,[^37^, ^38^] Figure 3 b: NRPS‐5, Figure S3) and the recombinant thiazole‐peptide (**6**)‐producing NRPS (RtpS,^[27]^ Figure 3 b: NRPS‐6, Figure S4). Whereas GxpS originates from the Gram‐negative bacterium *Photorhabdus luminescens* TT01, RtpS was constructed previously^[27]^ from building blocks of Gram‐positive origin (using NRPSs for the production of bacitracin^[39]^ and surfactin^[40]^). Both resulting type S NRPSs (Figure 3 b) showed good to very good titres of the desired peptides. NRPS‐5 produced **2** (Figure S3) with yields of ≈64 % (4.9 mg L^−1^) compared to WT GxpS and NRPS‐6 produced **6** (Figure S4) at WT RtpS level (≈20 mg L^−1^).

All aforementioned and following product structures and yields were confirmed by tandem mass spectrometry (MS/MS) analysis and comparison of the retention times with synthetic standards (see supplementary information).

### Bio‐Combinatoric Potential of Bipartite Type S NRPSs

Having a tool at hand to generate type S NRPSs that mimic WT behaviour, inter alia, the following questions arise: (Q1) Can type S NRPSs also be efficiently used for NRPS bio‐engineering purposes to produce natural product like peptide derivatives, as we have shown in previous work for the XU concept;^[19]^ and (Q2) how do SZs impact peptide production compared to covalently fused NRPSs?

**Figure 4:**
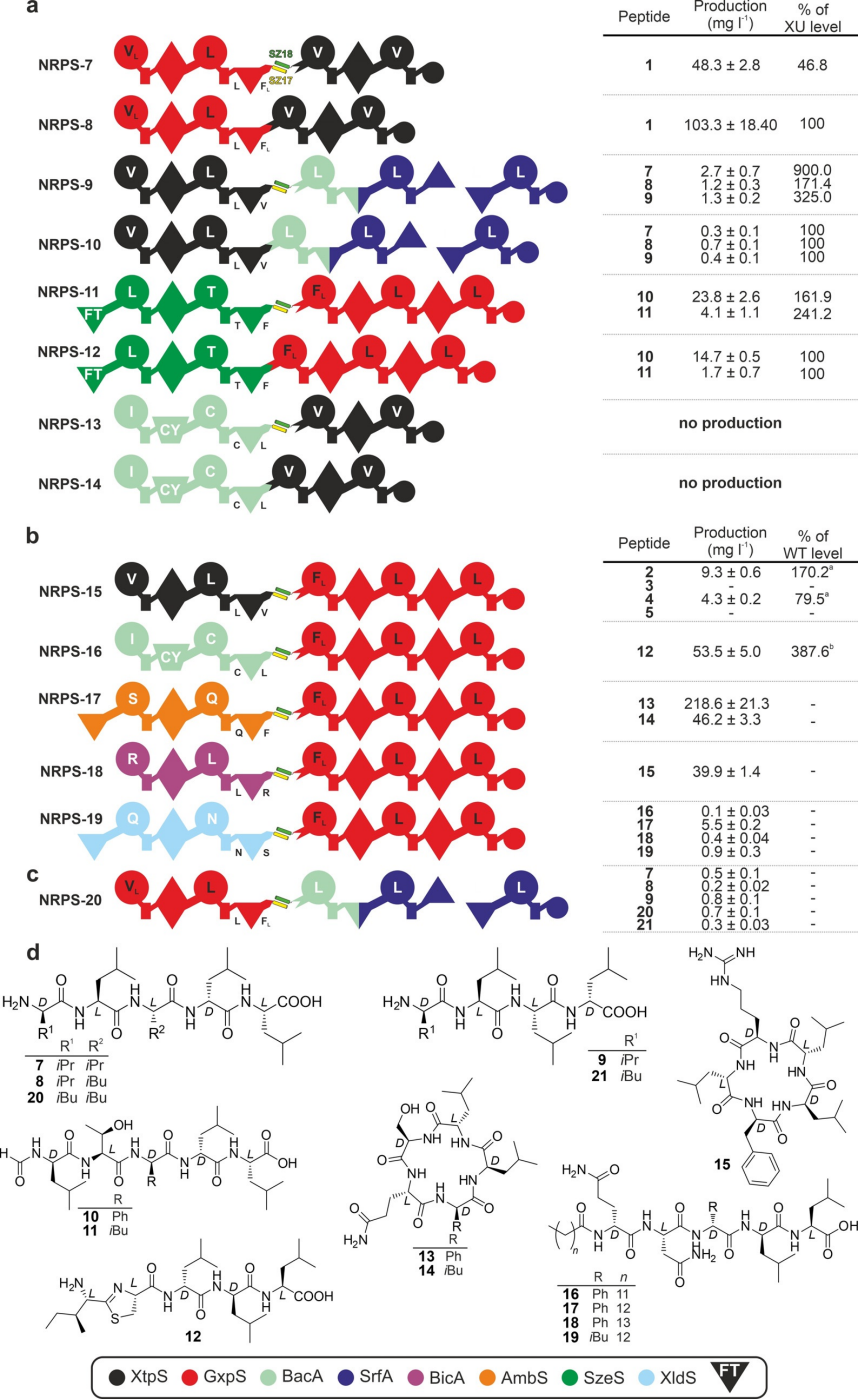
Bio‐combinatoric potential of bipartite type S NRPS. a) Production yields of type S NRPSs compared to analogous recombinant covalently fused NRPSs (**NRPS‐7** to **NRPS‐14**). b/c) Further type S NRPSs, each generated by co‐expressing two individual subunits (**NRPS‐15**–**NRPS‐20**). For NRPS‐15 & ‐16 production levels were compared to heterologously expressed WT BGCs of GxpS (^a^) and RtpS (^b^), respectively. For additional type S NRPS including non‐functional examples see Figure S17. d) The structures of **7**–**21** produced from **NRPS‐7** to **NRPS‐20** expressed in *E. coli*. See Figure 1 and 3 for assignment of the domain symbols; further symbols: FT, formyl‐transferase domain. The native C domains’ donor and acceptor site substrate specificity is given below selected C domains.

To answer these questions, we generated and co‐expressed four sets of synthetic NRPSs (Figure 4 a, NRPS‐7 – ‐14) by recombining building blocks from XtpS,^[34]^ GxpS,[^37^, ^38^] RtpS,^[27]^ and the szentiamide^[41]^‐producing synthetase (SzeS^[42]^), analysed the culture extracts by HPLC‐MS/MS and compared the resulting peptide titres. Each set consisted of two NRPSs: one type S NRPS, co‐expressing two non‐cognate subunits (NRPS‐7, ‐9, ‐11, ‐13); and a corresponding covalently fused NRPS version (NRPS‐8, ‐10, ‐12,^[19]^ ‐14), constructed according to the XU approach and largely in line with the XUs’ specificity rule to prevent potential substrate specificity issues[^19^, ^22^] at the upstream C domains’ acceptor site.

In sum, six out of eight recombinant NRPSs were functional (NRPS‐7 – ‐12; Figure 4 a). Whereas type S NRPS‐7 showed moderately decreased (≈47 %) yields of **1** compared to the covalently fused NRPS‐8, type S NRPS‐9 & ‐11 even showed 1.6‐ to 9‐fold increased productivity of peptide derivatives **7**–**9** and **10**–**11** (Figure 4 d, Figure S5–S10), respectively, compared to their covalent counterparts NRPS‐10 & ‐12. Only in case of type S NRPS‐13, but also for its covalent version NRPS‐14, no production was detected, suggesting a reason other than the SZs for their inactivity, likely being a result of the XtpS TE domain substrate specificity (*cf*. Figure S17).

Therefore, to a first approximation, it can be concluded that type S NRPSs can indeed be used for bioengineering and ‐combinatorial purposes (Q1) without impairing NRP biosynthesis more than recombinant *in cis* NRPSs (Q2) do anyway. A potential advantage is highlighted by type S NRPS‐9 (subunit 1: XtpS; subunit 2: RtpS) and its *in cis* variant NRPS‐10 (XU 1–2: XtpS; XU 3–5: RtpS). Both, NRPS‐9 and ‐10, are producing peptides **7**–**9** and are composed of building blocks from Gram‐negative (*X. nematophila* HGB081) and ‐positive (*B. licheniformis* ATCC 10716, *B. subtilis* ATCC 21332) origin. Whereas NRPS‐10 exhibits the typical impaired biosynthesis when XUs from Gram‐negative and ‐positive origin are combined,[^19^, ^27^] i.e., synthesising **7** (linear *v*LL*l*L; *D*‐AAs in italics and lowercase throughout this work) in yields of 0.3 mg L^−1^, type S NRPS‐9 shows a 9‐fold increased titre of **7** (2.7 mg L^−1^), indicating that observed impairments might be caused at the level of translation, rather than the proteins themselves. Nonrelated bacterial phyla, like *Proteobacteria* and *Firmicutes*, adopted varying codon usages imposing dares during protein translation (distribution of rare codons to control pace of translation and proper protein folding), even aggravated by the respective heterologous host. Separating translation of non‐related building blocks by introducing SZs mediated *in trans* protein‐protein communication may have minimized the effects of divergent codon usage on protein translation.

### Reuse of NRPS‐Encoding Plasmids Leads to Rapid Generation of Synthetic NRPSs

Type S NRPSs not only open the possibility to convert single protein type A NRPSs into separately expressible subunits (Figure 1 b, Figure 2, & Figure 3), but also to the reuse (Figure 4 a: NRPS‐7, ‐9, & ‐13) of already generated NRPS encoding plasmids. The reuse of these plasmids therefore opens the door to quickly generate a plethora of artificial BGCs from a small set of subunits by simple combinatorics. Thus, type S NRPSs might provide a means to overcome one of the major limiting factors in NRPS research and engineering efforts, namely the substantial amount of lab work involved in generating artificial BGCs (Figure 1 a).

To showcase possible applications, in a first step we created three additional type S subunits 1 (Figure 4 b, NRPS‐17 – ‐19) from XUs 1–2 of NRPSs producing ambactin (AmbS,^[35]^ *X. miraniensis* DSM 17902), bicornutin (BicA,^[43]^ *X. budapestensis* DSM 16342), and xenolindicin (XldS,^[35]^ *X. indica* DSM 17382). These subunits 1 (Figure 4 b) were selected to provide a certain variety of substrate specificity at the second (*C*‐terminal) XUs’ C domain acceptor site. Next, all subunits 1 were co‐expressed with all subunits 2. In total, 18 type S NRPSs were generated, and culture extracts analysed via HPLC‐MS/MS (Figure 4 a: NRPS‐7, ‐9, ‐11, ‐13; Figure 4 b: NRPS‐15 – ‐19; Figure 4 c: NRPS‐20; Figure S17: NRPS‐21 – ‐28).

In brief, 9 out of 18 type S NRPSs showed catalytic activity (Figure 4 a & b, Figure S17), synthesising 21 different linear‐ (**7**–**12**, **16**–**21**, Figure S7, S9, S12, S15, S16), cyclic‐ (**1**–**5**, **13**–**15**, Figure S5, S11, S13, S14), lipo‐ (**16**–**19**, Figure S15), formyl‐ (**10**, **11**, Figure S9), and thiazoline (**12**, Figure S12) containing peptides (**1**–**21**; Figure 3 c & 4 c) in yields ranging from ≈0.1–220 mg L^−1^. From these results it immediately became apparent that a large number of chimeric NRPSs and associated NRPs could be generated in no time, and with only a minimum of necessary wet lab work. Moreover, from a detailed analysis of the functional and non‐functional NRPS systems in Figure 4 and Figure S17, the following can be concluded:

All type S NRPSs sharing the same subunit 2 originating from GxpS (NRPS‐5) were functional (Figure 4 a: NRPS‐11, Figure 4 b: NRPS‐15 – ‐19, Figure 4 c: NRPS‐20), independent of subunit 1’s origin (Gram‐negative/‐positive) and/or C domains’ acceptor site substrate specificity. For instance, NRPS‐18 (BicA subunit 1 + GxpS subunit 2) and ‐19 (XldS subunit 1 + GxpS subunit 2), not complying with the XUs’ specificity rules (Figure 4 b), produced peptides **15** (≈40 mg L^−1^, Figure S14) and **16**–**19** (0.1–5.5 mg L^−1^, Figure S15), respectively. The latter peptides (**16**–**19**) only differ in the *N*‐terminal acyl starter unit, originating from the *E. coli* fatty‐acid pool, as also observed in the original xenolindicins.^[35]^ Especially NRPS‐18 was expected to be inactive, as previous studies have shown that the BicA C3 domain's acceptor site is highly specific for Arg and cannot process Phe or Leu when covalently fused to subunit 2.^[27]^ This might indicate that splitting *in cis* NRPSs in between C and A domains potentially decreases C domains’ acceptor site specificity by introducing more geometric flexibility and minimizing potentially restrictive effects on A domain movements.^[44]^ This finding in turn supports the idea that C domains indeed do not exhibit intrinsic substrate specificities, as suggested also by a recently published study.^[23]^ Nevertheless, the effects of C domains on the substrate activation profile of A domains can be observed using NRPS‐16 as an example. NRPS‐16 produced the linear thiazoline containing peptide IC*llL (**12**; ≈53 mg L^−1^; Figure S12). In its natural NRPS context as well as in vitro, the A3 domain of GxpS prefers Phe over Leu.^[27]^ In case of NRPS‐16, the terminal C domain of subunit 1, expecting Leu at its acceptor site, either prevents the incorporation of Phe due to its gatekeeping activity or rather fine tunes the downstream A domain's specificity. Similar effects of engineered NRPSs, exhibiting chimeric C‐A interfaces[^19^, ^44^] or C domains,^[27]^ have been described.

In contrast, all except one (NRPS‐7) type S NRPSs sharing subunit 2 from XtpS (NRPS‐1), did not produce detectable amounts of any peptide (Figure S17). In light of all co‐expression experiments (Figure 3, 4, S17), providing evidence that NRPS‐1 subunit 2 and all subunits 1 are functional, only one possible explanation remains—the respective TE domains high specificity for peptide length and/or amino acid composition. Catalytic activity of NRPS‐7 (Figure 4 a) confirms this. Subunits 1 of NRPS‐1 and NRPS‐7 possess synonymous A domain specificities, leading to the production of **1** in both cases (NRPS‐1 & ‐7), and thus preventing TE domain substrate specificity issues.

Reusing type S subunits also allows the functional co‐expression of building blocks from Gram‐negative and ‐positive origin. Albeit only 3 (NRPS‐9, ‐16, ‐20) out of 8 combinations (Figure S17) showed catalytic activity, type S NRPSs represent a first very quick strategy to co‐express different subunits of various origin with each other to identify functional combinations. For example, NRPS‐16 (subunit 1: Gram‐positive; subunit 2: Gram‐negative) produced **12** in yields of 54 mg L^−1^ and even exceeded WT production rates of GxpS as well as NRPS‐1.

### Unpaired Activity of GxpS Subunit 2

All type S NRPS split in between C‐A domains and sharing GxpS subunit 2 (NRPS‐5, ‐11, and NRPS‐15 – ‐19) showed an unexpected behaviour, producing a range of tripeptides (**22**/**23** and **24**/**25**) at high titre up to 86 mg L^−1^ related to the unpaired activity of GxpS subunit 2 (Figure S18). Due to the promiscuous GxpS A3 domain, **22**/**23** and **24**/**25** differ from each other at position one, either carrying Phenylalanine or Leucine. In addition, **22** and **24** show a *D*‐*D*‐*L* configuration, whereas **23** and **25** have a *L‐D*‐*L* configuration. Apart from being unfavourable when it comes to bio‐synthetic production of specific bioactive compounds for pharmaceutical application, this finding suggests that it is possible to repurpose elongation modules to initiate peptide biosynthesis—at least under certain conditions—as most recently reported in an in vitro study.^[45]^

## Conclusion

Recently the successful application of SZs to replace naturally present DDs in polyketide synthases (PKS) as a tool to create chimeric PKSs was published.^[31]^ Here we reported the use of high‐affinity SZs to split native single protein NRPSs into two individual subunits by introducing them at the previously described fusion point of the XU concept.^[19]^ Thus, artificially *in trans* regulated assembly lines were generated not only representing a new NRPS architecture, referred to as type S, but also proves to be extremely easy to handle, productive with WT level yields, and provides an unprecedented degree of recombinability. Having SZs at hand, peptide libraries quickly can be constructed with high success rates even when based on NRPSs originating from different strains or phyla. It might also be conceivable to combine different biosynthetic pathways co‐occurring in one strain in situ, by introducing SZs at the genomic level. We are convinced that further research into this direction, like elucidating structures of SZ connected NRPS domains, eventually will bring up even more versatile artificial DDs.

Finally, we believe that SZs will greatly accelerate NRPS research—not only for engineering purposes, but also for the in vivo characterization of domain and module specificities (e.g., focusing on C and TE domains). In particular, the ability to build plasmid libraries holds enormous potential and will take future bio‐combinatoric approaches to the next level, e.g., for early drug discovery and high‐throughput lead identification approaches.

## Conflict of interest

Goethe University has filed a patent on this NRPS engineering technology. The patent is currently pending.

## Supporting information

As a service to our authors and readers, this journal provides supporting information supplied by the authors. Such materials are peer reviewed and may be re‐organized for online delivery, but are not copy‐edited or typeset. Technical support issues arising from supporting information (other than missing files) should be addressed to the authors.

Supporting Information
