# Supplementary material for: Synthetic Zippers as an Enabling Tool for Engineering of Non‐Ribosomal Peptide Synthetases
Source: Angew Chem Int Ed Engl. 2021 Jun 27;60(32):17531–8. doi: 10.1002/anie.202102859 (PMC8362031; doi:10.1002/anie.202102859)
Supplement: Supplementary file 1 — Supporting Information [file ANIE-60-17531-s001.pdf]

Angewandte Chemie
Eine Zeitschrift der Gesellschaft Deutscher Chemiker

Supporting Information

Synthetic Zippers as an Enabling Tool for Engineering of Non-Ribosomal Peptide Synthetases**

*Kenan A. J. Bozhueyuek[+], Jonas Watzel[+], Nadya Abbood[+], and Helge B. Bode**

anie_202102859_sm_miscellaneous_information.pdf

Supplementary Information - Table of Contents

1 Material and methods 3

1.1 Cultivation of strains 3

1.2 Cloning of biosynthetic gene clusters 3

1.3 Heterologous expression of NRPS templates and LC-MS analysis 4

1.4 Peptide quantification 5

1.5 Chemical synthesis 5

2 Supplementary Tables 6

Table S1. ESI-MS data of all produced peptides. 6

Table S2. Strains used in this work. 7

Table S3. Plasmids used in this work. 7

Table S4. Oligonucleotides used in this work. 8

3 Supplementary Figures 10

Figure S1. A schematic representation of the xenotetrapeptide (1) producing type A NRPS (XtpS). 10

Figure S2. HPLC/MS data refers to Figure 3a (WT XtpS, NRPS-1 and -3) of compound 1 produced in *E. coli* DH10B::*mtaA*. 10

Figure S3. HPLC/MS data refers to Figure 3b (WT GxpS: dark colours, NRPS-5: pale colours) of compounds 2–5 (WT GxpS/NRPS-5), 22/23 (NRPS-5) and 24/25 (NRPS-5) produced in *E. coli* DH10B::*mtaA*. 11

Figure S4. HPLC/MS data refers to Figure 3b (WT RtpS: dark colours, NRPS-6: pale colours) of compound 6 produced in *E. coli* DH10B::*mtaA*. 12

Figure S5. HPLC/MS data refers to Figure 4a (NRPS-7) of compound 1 produced in *E. coli* DH10B::*mtaA*. 12

Figure S6. HPLC/MS data refers to Figure 4a (NRPS-8) of compound 1 produced in *E. coli* DH10B::*mtaA*. 13

Figure S7. HPLC/MS data refers to Figure 4a (NRPS-9) of compounds 7, 8 and 9 produced in *E. coli* DH10B::*mtaA*. 13

Figure S8. HPLC/MS data refers to Figure 4a (NRPS-10) of compounds 7, 8 and 9 produced in *E. coli* DH10B::*mtaA*. 14

Figure S9. HPLC/MS data refers to Figure 4a (NRPS-11) of compounds 10, 11, 23 and 24/25 produced in *E. coli* DH10B::*mtaA*. 15

Figure S10. HPLC/MS data refers to Figure 4a (NRPS-12) of compounds 10 and 11 produced in *E. coli* DH10B::*mtaA*. 16

Figure S11. HPLC/MS data refers to Figure 4b (NRPS-15) of compounds 2 and 4 produced in *E. coli* DH10B::*mtaA*. 16

Figure S12. HPLC/MS data refers to Figure 4b (NRPS-16) of compounds 22/23, 24/25 and 12 produced in *E. coli* DH10B::*mtaA*. 17

Figure S13. HPLC/MS data refers to Figure 4b (NRPS-17) of compounds 13, 14, 23 and 24/25 produced in *E. coli* DH10B::*mtaA*. 18

Figure S14. HPLC/MS data refers to Figure 4b (NRPS-18) of compounds 23, 25 and 15 produced in *E. coli* DH10B::*mtaA*. 19

Figure S15. HPLC/MS data refers to Figure 4b (NRPS-19) of compounds 16–19 and 25 produced in *E. coli* DH10B::*mtaA*. 20

Figure S16. HPLC/MS data refers to Figure 4c (NRPS-20) of compounds 7, 8, 20, 9 and 21 produced in *E. coli* DH10B::*mtaA*. 21

Figure S17. A schematic representation of all bipartite type S NRPSs 23

Figure S18. (a) Production of *D*/*L*-tripeptides exemplary of NRPS-5........ 24

4 References........ 25

1 Material and methods

1.1 Cultivation of strains

All *E. coli*, *Xenorhabdus* and *Photorhabdus* strains were cultivated in liquid or on solid LB-medium (pH 7.5, 10 g/L tryptone, 5 g/L yeast extract and 5 g/L NaCl). Solid media contained 1% (w/v) agar. Kanamycin (50 µg/ml) and chloramphenicol (34 µg/ml) were used as selection markers. All *E. coli* cultures were cultivated at 37 °C and at 22 °C for peptide production purposes. *Xenorhabdus* and *Photorhabdus* strains were grown at 30 °C.

1.2 Cloning of biosynthetic gene clusters

Genomic DNA of selected *Xenorhabdus* and *Photorhabdus* strains were isolated using the Qiagen Gentra Puregene Yeast/Bact Kit. All PCRs were performed with oligonucleotides obtained from Eurofins Genomics (Supplementary Table 4). NRPS fragments for Hot Fusion cloning[1] were amplified with primers coding for the respective homology arms (20–30 bp) in a two-step PCR program. The coding sequences for the SYNZIPs were also attached upstream or downstream to the NRPS genes by PCR. In the following, the cloning procedure for the basic vectors is explained. pJW61/62 was obtained by the following steps: First, the SYNZIP17/18 coding sequences (pENTR-SYNZIP17/18[2] were a gift from Amy Keating, Addgene plasmids #80671/80672; RRID:Addgene_80671/80672) were inserted into the plasmids pCOLA_ara/tacI and pCK_0402 by oligonucleotides KB-pACYC-FW/RV or KB-pCOLA-FW/RV in two-step polymerase chain reactions (PCRs) combined with Hot Fusion Cloning[1]. Second, these plasmids were linearized by single-step PCRs with the help of the oligonucleotides KB-pCOLA-II-FW/RV or KB-pACYC-II-FW/RV, which further allowed us to introduce NRPS fragments by Hot Fusion cloning. Therefore, the respective NRPS coding sequences were amplified again in two-step PCRs, using oligonucleotides with additional coding regions for homology arms (20–30 bp). pJW63/64 coding for subunits of the XtpS without attached SYNZIPs were generated by amplifying pJW61/62 with a single phosphorylated [phos.] oligonucleotide pair excluding the SYNZIP coding region followed by T4 DNA ligation (following Thermo Fisher manufacturers' instructions). The control plasmids pCOLA_ara_xtpS/gxpS_tacI_JW coding for the native single protein xtpS/ gxpS were created by Hot Fusion Cloning. Therefore, the plasmid pCOLA_ara/tacI was linearized

by PCR using the oligonucleotides AL-XtpS-2-1 and AD64 and the insert *xtpS* was PCR amplified with the oligonucleotides jw0136_FW and jw0137_RV.

The plasmid pCOLA_ara_ gxpS_tacI_JW was generated in two Hot Fusion Cloning steps. First, the pCOLA_ara/tacI was linearized by PCR using the primers JW_tacI_PstI_FW2 and jw0064_RV and second the first part of *gxpS* was amplified using the oligonucleotides jw0124_FW/jw0160_RV. This intermediate plasmid was then opened with PstI and the second *gxpS* part, amplified with jw0151_FW/ jw0161_RV by PCR, was then integrated into the cleaving site by Hot Fusion Cloning. In all PCRs the S7 Fusion High-Fidelity DNA Polymerase (Mobidiag) was used according to the manufacturers' instructions. The amplified DNA was purified with the Invisorb Fragment CleanUp or MSB Spin PCRapace Kits (stratec molecular). The basic cloning of all new generated plasmids (Supplementary Table 3) was performed in *E. coli* DH10B. Each NRPS (subunit) was under the control of a P_{BAD} promotor. Plasmid isolation from *E. coli* was achieved with the Invisorb Spin Plasmid Mini Two Kit (stratec molecular). Restriction enzyme digests and the partial sequencing of essential plasmid regions especially upstream and downstream of the NRPS genes, where the SYNZIP coding sequences were located, confirmed the correct plasmid construction.

1.3 Heterologous expression of NRPS templates and LC-MS analysis

Constructed plasmids were transformed into *E. coli* DH10B::*mtaA*. Cells were grown overnight in LB medium containing the necessary antibiotics (50 µg/ml kanamycin, 34 µg/ml chloramphenicol). 100 µl of an overnight culture were used for inoculation of 10 ml LB-cultures supplemented with the respective antibiotics as selection markers and additionally containing 0.002 mg/ml L-arabinose and 2 % (v/v) XAD-16. After incubation for 72 h at 22 °C the XAD-16 was harvested. One culture volume methanol was added and incubated for 60 min at 22 °C. The organic phase was filtrated and a sample was taken of the cleared extract. After centrifugation (17,000 x *g*, 20 min) the methanol extracts were used for LC-MS analysis. All measurements were performed by using an Ultimate 3000 LC system (Dionex) with an ACQUITY UPLC BEH C18 column (130 Å, 2.1 x 50 mm, 1.7 µm particle size; Waters) at a flow rate of 0.4 ml min^{-1} using acetonitrile (ACN) and water containing 0.1% formic acid (v/v) in a gradient ranging from 5–95% of ACN over 16 min (40 °C) coupled to an AmaZonX (Bruker) electron spray ionization mass spectrometer. The BPC spectra were recorded in

positive ion mode with a mass range from 100–1200 *m/z* and ultraviolet (UV) wavelength range from 200–600 nm. The software Compass DataAnalysis 4.3 (Bruker) was used to evaluate the measurements.

1.4 Peptide quantification

The absolute production titers of selected peptides were calculated with calibration curves based on pure synthetic **1**, **2** (for quantification of **2–5**), **6** (for quantification of **6** and **12**), **7** (for quantification of **7, 8** and **20**), **9** (for quantification of **9**, **21**), **10** (for quantification of **10** and **11**), **13** (for quantification of **13** and **14**), **15**, **17** (for quantification of **16**–**19**) and **24** (for quantification of **22/23** and **24/25**). Therefore, the pure compounds were prepared at different concentrations (100, 50, 25, 12.5, 6.25, 3.125, 1.56, 0.78, 0.39, 0.195 and 0.0195 µg/ml) and measured by LC-MS using HPLC/MS measurements as described above. The peak area for each compound at different concentrations was calculated using Compass Data Analysis and used for the calculation of a standard curve passing through the origin. Triplicates of all *in vivo* experiments were measured. The pure peptide standards **1**, **2**, **6, 7**, **9**, **10**, **13**, **17** and **24** were synthesized in-house[3,4] and the further pure synthetic **15** was produced by Synpeptide.

1.5 Chemical synthesis

Chemical synthesis of all peptides was performed as described previously[3].

2 Supplementary Tables

Table S1. ESI-MS data of all produced peptides.

Peptide (#)	theoretical mass-to-charge ratio (*m/z*) [M+H]$^+$	Molecular formula	Reference
**1**	410.29	$C_{21}H_{38}N_4O_4$	[5]
**2**	586.40	$C_{32}H_{51}O_5N_5$	[6]
**3**	600.41	$C_{33}H_{53}O_5N_5$	[6]
**4**	552.41	$C_{29}H_{53}O_5N_5$	[6]
**5**	566.43	$C_{30}H_{55}O_5N_5$	[6]
**6**	556.35	$C_{27}H_{49}N_5O_5S$	-
**7**	556.41	$C_{28}H_{53}N_5O_6$	-
**8**	570.42	$C_{29}H_{55}N_5O_6$	-
**9**	457.34	$C_{23}H_{44}N_4O_5$	-
**10**	634.38	$C_{32}H_{51}N_5O_8$	[3]
**11**	600.40	$C_{29}H_{53}N_5O_8$	[3]
**12**	556.35	$C_{27}H_{49}N_5O_5S$	-
**13**	589.33	$C_{29}H_{44}N_6O_7$	-
**14**	555.35	$C_{26}H_{46}N_6O_7$	-
**15**	643.43	$C_{33}H_{54}N_8O_5$	-
**16**	830.54	$C_{43}H_{71}N_7O_9$	-
**17**	844.55	$C_{44}H_{73}N_7O_9$	-
**18**	858.57	$C_{45}H_{75}N_7O_9$	-
**19**	810.57	$C_{41}H_{75}N_7O_9$	-
**20**	584.44	$C_{30}H_{57}N_5O_6$	-
**21**	471.35	$C_{24}H_{46}N_4O_5$	-
**22**	358.27	$C_{18}H_{35}N_3O_4$	-
**23**	358.27	$C_{18}H_{35}N_3O_4$	-
**24**	392.25	$C_{21}H_{33}N_3O_4$	-
**25**	392.25	$C_{21}H_{33}N_3O_4$	-

Table S2. Strains used in this work.

Strain	Genotype/NRPS	Reference
E. coli DH10B	F_mcrA (*mrr-hsd*RMS-*mcr*BC), 80*lac*ZΔ, M15, Δ*lac*X74 *rec*A1 *end*A1 *ara*D 139Δ(*ara*, *leu*)7697 *gal*U *gal*K λ *rps*L (*Strr*) *nup*G	[7]
E. coli DH10B::*mtaA*	DH10B with *mtaA* from pCK_*mtaA*Δ*entD*	[8]
P. luminescens TTO1	*gxpS*[6]	DSMZ
X. nematophila ATCC 19061	*xtpS*[5]	ATCC
X. budapestensis DSM 16342	*bicA*[9]	DSMZ
X. miraniensis DSM 17902	*ambS*[8]	DSMZ
X. szentirmaii DSM16338	*szeS*[10]	DSMZ
X. indica DSM 17382	*xldS*[8]	DSMZ
B. licheniformis ATCC 10716	*bacA*[11]	M. A. Marahiel / ATCC
B. subitlis MR 168	*srfA*[12]	ATCC

Table S3. Plasmids used in this work.

Plasmids	Genotype	Reference
pFF1_22A_szeS_gxpS (NRPS-12)	ori 2µ, kanMX4, ori ColA, kan^R, *P*$_{BAD}$ *szeS*_Ft$A_1T_1C/E_2A_2T_2C_3$-*gxpS*_$A_3T_3C/E_4A_4T_4C/E_5A_5T_5$TE, Ypet-Flag	[3]
pFF1_NRPS_6	ori 2µ, kanMX4, *araC-P*$_{BAD}$, ori ColA, Ypet-Flag, kan^R, *bacA*-A1T1CyA2T2C3A3T3CD$_{sub4}$_*sfrA-BC*-C$_{Asub6}$A6T6E6C7A7T7TE	[4]
pCOLA_ara/tacI	ori ColA, kan^R, *araC-P*$_{BAD}$ and *tac*I	[13]
pCK_0402	ori p15A, cm^R, *araC-P*$_{BAD}$ and *tac*I-*araE*	[14]
pCOLA_ara_xtpS_tacI_JW	ori ColA, kan^R, *araC-P*$_{BAD}$ *xtpS* and *tac*I	this study
pCOLA_ara_gxpS_tacI_JW	ori ColA, kan^R, *araC-P*$_{BAD}$ *gxpS* and *tac*I	this study
pJW61	ori p15A, cm^R, *araC-P*$_{BAD}$ *xtpS*_$A_1T_1C/E_2A_2T_2C_3$-SYNZIP17 and *tac*I-*araE*	this study
pJW62	ori ColA, kan^R, *araC-P*$_{BAD}$ SYNZIP18-*xtpS*_$A_3T_3C/E_4A_4T_4$TE and *tac*I	this study
pJW63	ori p15A, cm^R, *araC-P*$_{BAD}$ *xtpS*_$A_1T_1C/E_2A_2T_2C_3$ and *tac*I-*araE*	this study
pJW64	ori ColA, kan^R, *araC-P*$_{BAD}$ *xtpS*_$A_3T_3C/E_4A_4T_4$TE, *tac*I	this study
pJW75	ori p15A, cm^R, *araC-P*$_{BAD}$ *gxpS*_$A_1T_1C/E_2A_2T_2C_3$-SYNZIP17 and *tac*I-*araE*	this study
pJW76	ori ColA, kan^R, *araC-P*$_{BAD}$ SYNZIP18-*gxpS*_$A_3T_3C/E_4A_4T_4C/E_5A_5T_5$TE and *tac*I	this study
pJW77	ori p15A, cm^R, *araC-P*$_{BAD}$ *bicA*_$A_1T_1C/E_2A_2T_2C_3$-SYNZIP17 and *tac*I-*araE*	this study
pJW91	ori p15A, cm^R, *araC-P*$_{BAD}$ *ambS*_ $A_1T_1C/E_2A_2T_2C_3$-SYNZIP17 and *tac*I-*araE*	this study
pJW92	ori p15A, cm^R, *araC-P*$_{BAD}$ *szeS*_Ft$A_1T_1C/E_2A_2T_2C_3$-SYNZIP17 and *tac*I-*araE*	this study
pJW93	ori p15A, cm^R, *araC-P*$_{BAD}$ *xldS*_$C_1A_1T_1C/E_2A_2T_2C_3$-SYNZIP17 and *tac*I-*araE*	this study
pJW114	ori p15A, cm^R, *araC-P*$_{BAD}$ *bacA*_$A_1T_1CyA_2T_2C_3$-SYNZIP17 and *tac*I-*araE*	this study
pJW116	ori ColA, kan^R, *araC-P*$_{BAD}$ SYNZIP18-*bacA*_$A_3T_3C_{Dsub4}$-*sfrA-BC*_$C_{Asub6}A_6T_6E_6C_7A_7T_7$TE and *tac*I	this study
pJW159	ori ColA, kan^R, *araC-P*$_{BAD}$ *xtpS*_ $A_1T_1C/E_2A_2T_2C_3$-*sfrA-BC*_$C_{Asub6}A_6T_6E_6C_7A_7T_7$TE and *tac*I	this study
pJW160	ori ColA, kan^R, *araC-P*$_{BAD}$ *bacA*_$A_1T_1CyA_2T_2C_3$-*xtpS*_$A_3T_3C/E_4A_4T_4$TE and *tac*I	this study
pJW161	ori ColA, kan^R, *araC-P*$_{BAD}$ *gxpS*_$A_1T_1C/E_2A_2T_2C_3$-*xtpS*_$A_3T_3C/E_4A_4T_4$TE and *tac*I	this study

Table S4. Oligonucleotides used in this work. Correlations of plasmids to figures from the main text and supplementary information are represented in brackets.

Plasmids	Oligo-nucleotide	Sequence (5´→3´; overlapping ends)	Template
pJW61 (NRPS-1, NRPS-2, NRPS-9, NRPS-15)	KB-pACYC-FW	GAACAGTTAAAACAGAAGCGTGAACAATTAAAGCAAAAGATCGCCAATCTGCGTAA GGAGATCGAAGCCTACAAGTGACAATTAATCATCGGCTCG	pCK_0402
	KB-pACYC-RV	TTCACGCTTCTGTTTTAACTGTTCGATGCGATTACGCAATTCAGCCTTTTTCGATTTT AATTCCTCCTTCTCGTTCATGGAATTCCTCCTGTTAGC	pCK_0402
	KB-pACYC-II-FW	AACGAGAAGGAGGAATTAAAATCG	-
	KB-pACYC-II-RV	CATGGAATTCCTCCTGTTAGC	-
	KB-P1-FW	TGGGCTAACAGGAGGAATTCCATGAAAGATAGCATGGCTAAAAAGGG	*X. nematophila* ATCC 19061
	KB-P1-RV	CGATTTTAATTCCTCCTTCTCGTTCCAGGTTTTTAACAACAATGTGC	*X. nematophila* ATCC 19061
pJW62 (NRPS-1, NRPS-3, NRPS-7, NRPS-13, NRPS-21, NRPS-22, NRPS-23, NRPS-24)	KB-pCOLA-FW	CATTGACAAAGAGCTGCGTGCCAACGAAAACGAACTTCGCGCCCTTGATAACGAGC TGACTGCAGCTATCTCATGACAATTAATCATCGGCTCG	pCOLA_ara/tacI
	KB-pCOLA-RV	TTGGCACGCAGCTCTTTGTCAATGGCATTTAACTCGCGGTCCAAGGCTTTCAGTTCA CGCTCTTCAGCATAGAACATGGAATTCCTCCTGTTAGC	pCOLA_ara/tacI
	KB-pCOLA-II-FW	TGACAATTAATCATCGGCTCG	-
	KB-pCOLA-II-RV	TGAGATAGCTGCAGTCAGCTCG	-
	KB-P2-FW	AACGAGCTGACTGCAGCTATCTCATTATGTATTCATCAACTTTTTGAACAGC	*X. nematophila* ATCC 19061
	KB-P2-RV	ATACGAGCCGATGATTAATTGTCACAGCGCCTCCACTTCG	*X. nematophila* ATCC 19061
pJW63 (NRPS-3, NRPS-4)	jw0061_FW	[phos.] TGACAATTAATCATCGGCTCG	pJW61
	jw0062_RV	CCAGGTTTTTAACAACAATGTGC	pJW61
pJW64 (NRPS-2, NRPS-4)	jw0063_FW	[phos.] TTATGTATTCATCAACTTTTTGAACAGC	pJW62
	jw0064_RV	CATGGAATTCCTCCTGTTAGCC	pJW62
pJW75 (NRPS-5, NRPS-7, NRPS-20)	KB-pACYC-II-FW	AACGAGAAGGAGGAATTAAAATCG	pJW61
	KB-pACYC-II-RV	CATGGAATTCCTCCTGTTAGC	pJW61
	jw0124_FW	GGGCTAACAGGAGGAATTCCATGAAAGATAGCATGGCTAAAAAGGAAATTATC	*P. luminescens* TTO1
	jw0125_RV	TCGATTTTAATTCCTCCTTCTCGTTCCAATTTTCCAGTAATAACTCCCG	*P. luminescens* TTO1
pJW76 (NRPS-5, NRPS-11, NRPS-15, NRPS-16, NRPS-17, NRPS-18, NRPS-19)	KB-pCOLA-II-FW	TGACAATTAATCATCGGCTCG	pJW62
	KB-pCOLA-II-RV	TGAGATAGCTGCAGTCAGCTCG	pJW62
	jw0172_FW	GGCTAACAGGAGGAATTCCATGTTCTATGCTGAAGAGCGTGAAC	*P. luminescens* TTO1
	jw0127_RV	CGAGCCGATGATTAATTGTCACAGCGCCTCCGCTTC	*P. luminescens* TTO1
pJW114 (NRPS-6, NRPS-13, NRPS-16)	KB-pACYC-II-FW	AACGAGAAGGAGGAATTAAAATCG	pJW61
	KB-pACYC-II-RV	CATGGAATTCCTCCTGTTAGC	pJW61
	jw208_FW	GCTAACAGGAGGAATTCCATGGTTGCTAAACATTCATTAGAAAATGGG	pFF1_NRPS_6[4]
	jw209_RV	CGATTTTAATTCCTCCTTCTCGTTCTTTGTATGGTTAAAGGACTCTAAAAGTGTC	pFF1_NRPS_6[4]
pJW116 (NRPS-6, NRPS-9, NRPS-10, NRPS-20, NRPS-25, NRPS-26, NRPS-27, NRPS-28)	KB-pCOLA-II-FW	TGACAATTAATCATCGGCTCG	pJW62
	KB-pCOLA-II-RV	TGAGATAGCTGCAGTCAGCTCG	pJW62
	jw0211_FW	CGAGCTGACTGCAGCTATCTCAAAAGCAATCCACCAGCTGTTT	pFF1_NRPS_6[4]
	jw0212_RV	CGAGCCGATGATTAATTGTCATGAAACCGTTACGGTTTGTGTATTA	pFF1_NRPS_6[4]
pJW77 (NRPS-18, NRPS-23, NRPS-27)	KB-pACYC-II-FW	AACGAGAAGGAGGAATTAAAATCG	pJW61
	KB-pACYC-II-RV	CATGGAATTCCTCCTGTTAGC	pJW61
	jw0128_FW	GGGCTAACAGGAGGAATTCCATGAAAGATAACATTGCTACAGTGGCAAATAG	*X. budapestensis* DSM 16342
	jw0129_RV	CGATTTTAATTCCTCCTTCTCGTTCCAAGTTTTCAGCAACAACTGG	*X. budapestensis* DSM 16342
pJW91 (NRPS-17, NRPS-21, NRPS-25)	KB-pACYC-II-FW	AACGAGAAGGAGGAATTAAAATCG	pJW61
	KB-pACYC-II-RV	CATGGAATTCCTCCTGTTAGC	pJW61
	jw0162_FW	GCTAACAGGAGGAATTCCATGAAAAATGATAAGGTGATGACTCTG	*X. miraniensis* DSM 17902
	jw0163_RV	TCGATTTTAATTCCTCCTTCTCGTTCCACGTTTCCAGCAATAACC	*X. miraniensis* DSM 17902
pJW92 (NRPS-11, NRPS-22, NRPS-26)	KB-pACYC-II-FW	AACGAGAAGGAGGAATTAAAATCG	pJW61
	KB-pACYC-II-RV	CATGGAATTCCTCCTGTTAGC	pJW61
	jw0164_FW	GCTAACAGGAGGAATTCCATGAAAGGTAGTATTGCTAAAAAGGGAG	*X. szentirmaii* DSM16338
	jw0165_RV	TCGATTTTAATTCCTCCTTCTCGTTCCAGCTTTCCAGCAATAACC	*X. szentirmaii* DSM16338
pJW93 (NRPS-19, NRPS-24, NRPS-28)	KB-pACYC-II-FW	AACGAGAAGGAGGAATTAAAATCG	pJW61
	KB-pACYC-II-RV	CATGGAATTCCTCCTGTTAGC	pJW61

	jw0166_FW	GCTAACAGGAGGAATTCCATGAAACTTTGGAACTATAAAATGAATATGAC	*X. indica* DSM 17382
	jw0167_RV	TCGATTTTAATTCCTCCTTCTCGTTGAAATCCACCAACAGTTGTTGAC	*X. indica* DSM 17382
pJW159 (NRPS-10)	KB-pCOLA-II-FW	TGACAATTAATCATCGGCTCG	pJW62
	jw0064_RV	CATGGAATTCCTCCTGTTAGCC	pJW62
	na_0194_FW	GGCTAACAGGAGGAATTCCATGAAAGATAGCATGGCTAAAAAGG	pJW61
	jw0284_RV	GCCGTCTTTGTATGGTTAAAGGTTTTTAACAACAATGTGCGTTC	pJW61
	jw0285_FW	TTTAACCATACAAAGACGGCATATCCAAAAGGAAAAGCAATCCACCAGCTGTTT	pJW116
	jw0212_RV	CGAGCCGATGATTAATTGTCATGAAACCGTTACGGTTTGTGTATTA	pJW116
pJW160 (NRPS-14)	KB-pCOLA-II-FW	TGACAATTAATCATCGGCTCG	pJW62
	jw0064_RV	CATGGAATTCCTCCTGTTAGCC	pJW62
	jw0208_FW	GCTAACAGGAGGAATTCCATGGTTGCTAAACATTCATTAGAAAATGGG	pJW114
	jw0286_RV	ACGGTTTCAGTGGCATTCCAGGACTCTAAAAGTGTCCGTTTTTCTTGAC	pJW114
	jw0287_FW	TGGAATGCCACTGAAACCGTGTATCCTGAATCGTTATGTATTCATCAACTTTTTGAA CAGC	pJW62
	jw0188_RV	GCCTAAACCAATACGCCGT	pJW62
	jw0189_FW	CGGCGTATTGGTTTAGGCCTGT	pJW62
	na07_RV	CGAGCCGATGATTAATTGTCACAGCGCCTCCACTTCG	pJW62
pJW161 (NRPS-8)	KB-pCOLA-II-FW	TGACAATTAATCATCGGCTCG	pJW62
	jw0064_RV	CATGGAATTCCTCCTGTTAGCC	pJW62
	jw0124_FW	GGGCTAACAGGAGGAATTCCATGAAAGATAGCATGGCTAAAAAGGAAATTATC	pJW75
	jw0288_RV	ACGGTTTCAGTGGCATTCCAATTTTCCAGTAATAACTCCCGCTCAG	pJW75
	jw_0287_FW	TGGAATGCCACTGAAACCGTGTATCCTGAATCGTTATGTATTCATCAACTTTTTGAA CAGC	pJW62
	jw0188_RV	GCCTAAACCAATACGCCGT	pJW62
	jw0189_FW	CGGCGTATTGGTTTAGGCCTGT	pJW62
	na07_RV	CGAGCCGATGATTAATTGTCACAGCGCCTCCACTTCG	pJW62
pCOLA_ara_xtpS _tacI_JW	jw0136_FW	CGCTGCTGGTTCTGGCGATTGACAATTAATCATCGGCTCG	pCOLA_ara/tacI
	jw0137_RV	AACGGGTATGGAGAAACAGTAGAGAGTTGCGATAAAAAGCG	pCOLA_ara/tacI
	AL-GxpS-2-1	ACTGTTTCTCCATACCCGTTTTTTGGGCTAACAGGAGGAATTCCATGAAAGATAGC ATGGCTAAAAAGG	*X. nematophila* ATCC 19061
	AD64	TCGCCAGAACCAGCAGCGGAGCCAGCGGATCCGGCGCGCCTTACAGCGCCTCCA C	*X. nematophila* ATCC 19061
pCOLA_ara_gxpS _tacI_JW	JW_tacI_PstI_FW 2	CTGCAGGAGCTGTTGACAAT	pCOLA_ara/tacI
	jw0064_RV	CATGGAATTCCTCCTGTTAGCC	pCOLA_ara/tacI
	jw0124_FW	GGGCTAACAGGAGGAATTCCATGAAAGATAGCATGGCTAAAAAGGAAATTATC	*P. luminescens* TTO1
	jw0160_RW	GATTAATTGTCAACAGCTCCTGCAGGCAGATAGAGACGTTTGTTGGC	*P. luminescens* TTO1
	jw0151_FW/	GCCAACAAACGTCTCTATCTGCTGGATGAACACCG	*P. luminescens* TTO1
	jw0161_RV	GATTAATTGTCAACAGCTCCTGCAGTCACAGCGCCTCCGCTTCAC	*P. luminescens* TTO1

3 Supplementary Figures

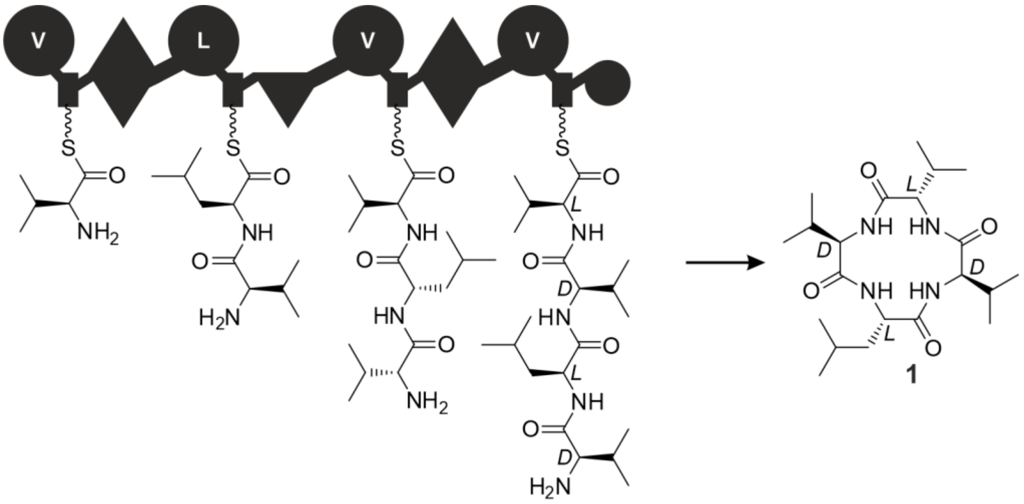

Figure S1. A schematic representation of the xenotetrapeptide (**1**) producing type A NRPS (XtpS). For domain assignment the following symbols are used: A, adenylation domain, large circles; T, thiolation domain, rectangle; C, condensation domain, triangle; C/E, dual condensation/epimerization domain, diamond; TE, thioesterase domain, small circle.

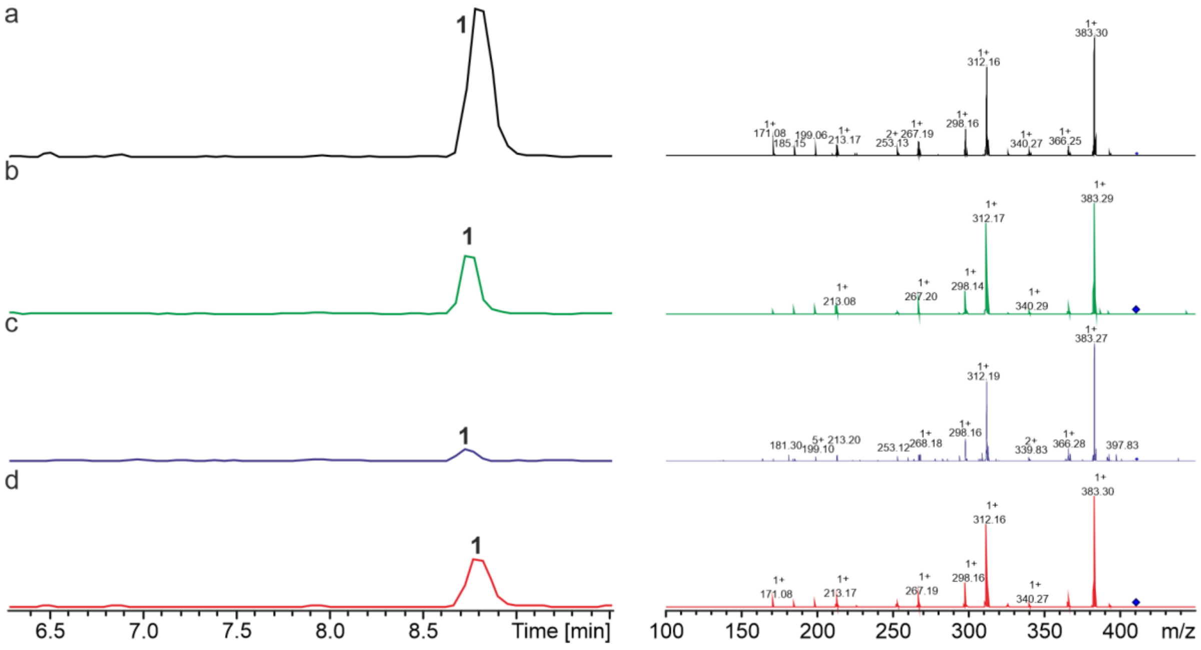

Figure S2. HPLC/MS data refers to Figure 3a (WT XtpS, NRPS-1 and -3) of compound **1** produced in *E. coli* DH10B::*mtaA*. (a) Extracted ion chromatogram (EIC)/MS^2 of **1** (m/z $[M+H]^+$ = 411.30; WT XtpS). (b) EIC/MS^2 of **1** (m/z $[M+H]^+$ = 411.30; NRPS-1). (c) EIC/MS^2 of **1** (m/z $[M+H]^+$ = 411.30; NRPS-3). EICs (a–c) are displayed with the same intensity range. (d) EIC/MS^2 data of synthetic **1** (m/z $[M+H]^+$ = 411.30).

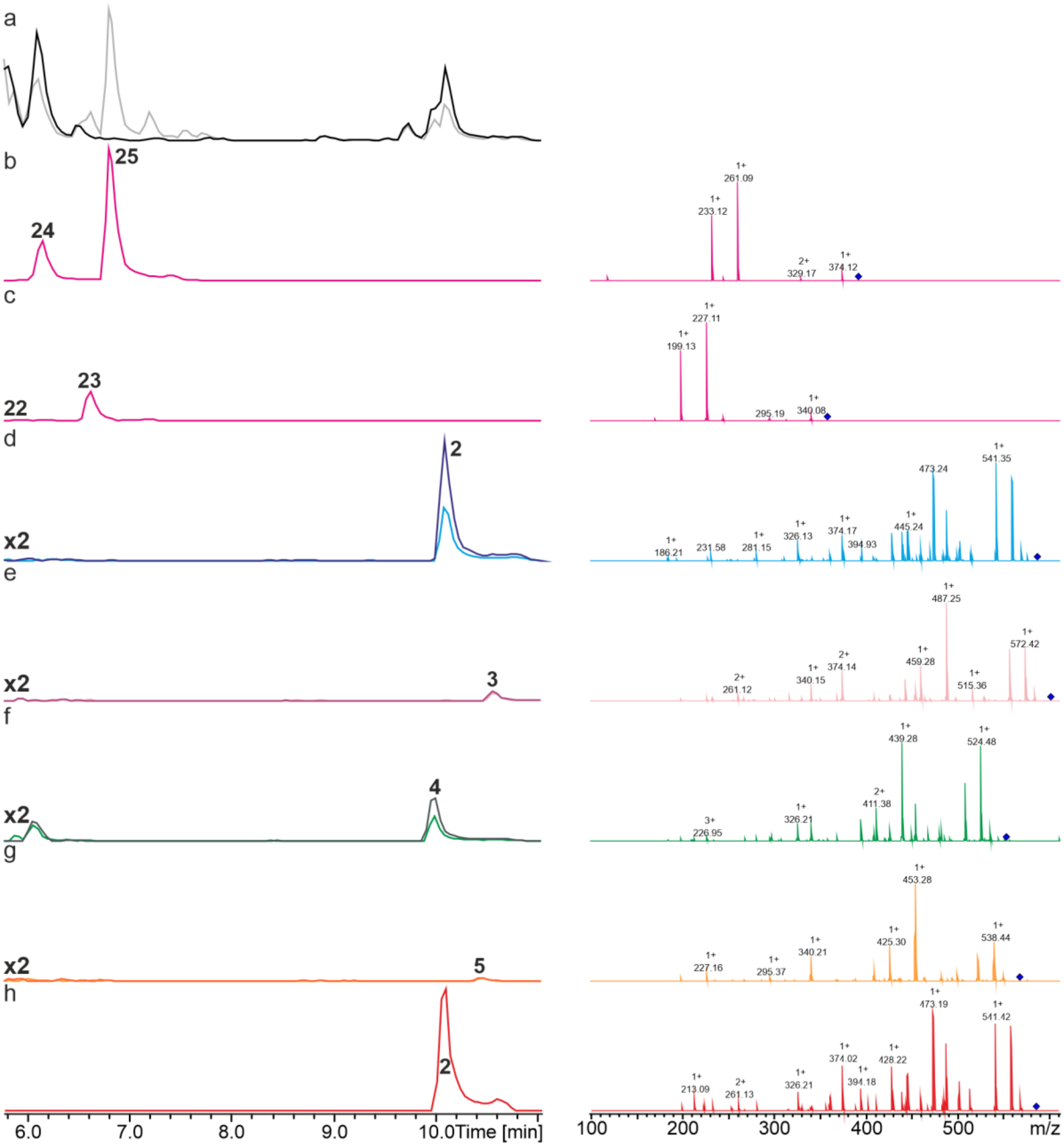

Figure S3. HPLC/MS data refers to Figure 3b (WT GxpS: dark colours, NRPS-5: pale colours) of compounds **2**–**5** (WT GxpS/NRPS-5), **22/23** (NRPS-5) and **24/25** (NRPS-5) produced in *E. coli* DH10B::*mtaA*. (a) Base Peak Chromatogram (BPC) of an exemplary culture extract. (b) EIC/MS^2 of **24/25** (*m/z* $[M+H]^+$ = 392.25). (c) EIC/MS^2 of **22/23** (*m/z* $[M+H]^+$ = 358.27). (d) EIC/MS^2 of **2** (*m/z* $[M+H]^+$ = 586.40). (e) EIC/MS^2 of **3** (*m/z* $[M+H]^+$ = 600.41). (f) EIC/MS^2 of **4** (*m/z* $[M+H]^+$ = 552.41). (g) EIC/MS^2 **5** (*m/z* $[M+H]^+$ = 566.43). BPCs/EICs (a–c) are displayed with the same intensity range, whereas EICs (d–g) of **2-5** are depicted with 2-fold increased intensity. (h) EIC/MS^2 of synthetic **2** (*m/z* $[M+H]^+$ = 586.40).

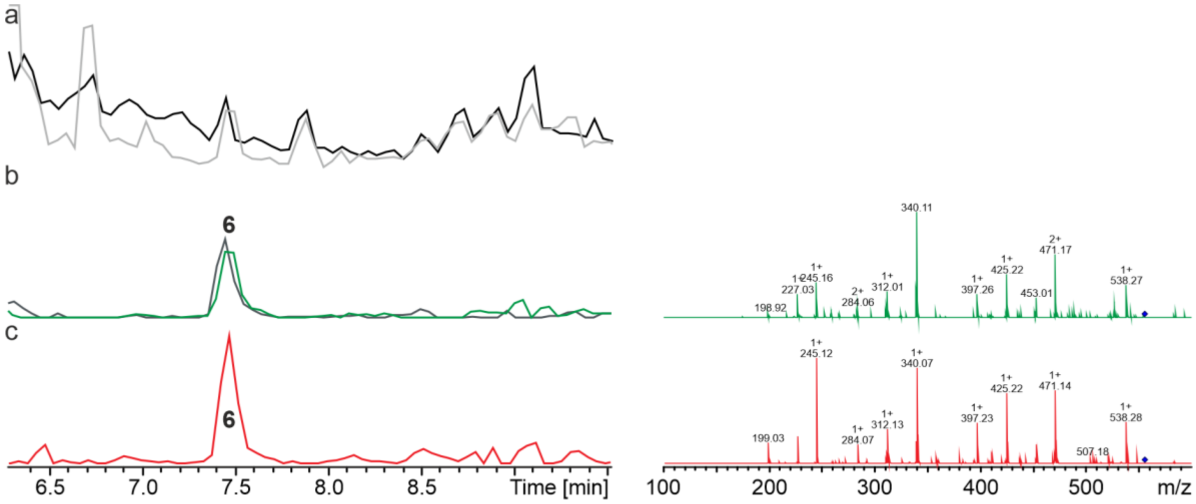

Figure S4. HPLC/MS data refers to Figure 3b (WT RtpS: dark colours, NRPS-6: pale colours) of compound **6** produced in *E. coli* DH10B::*mtaA*. (a) BPC of an exemplary culture extract. (b) EIC/MS^2 of **6** (*m/z* $[M+H]^+$ = 556.35). BPCs/EICs (a/b) are displayed with the same intensity range. (c) EIC/MS^2 of synthetic **6** (*m/z* $[M+H]^+$ = 556.35).

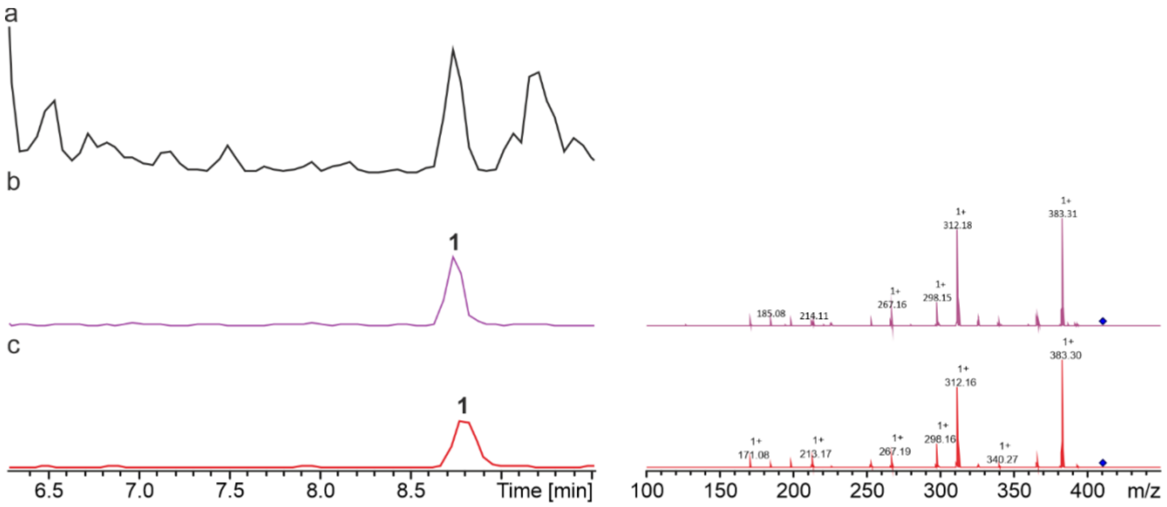

Figure S5. HPLC/MS data refers to Figure 4a (NRPS-7) of compound **1** produced in *E. coli* DH10B::*mtaA*. (a) BPC of an exemplary culture extract. (b) EIC/MS^2 of **1** (*m/z* $[M+H]^+$ = 411.30). (c) EIC/MS^2 of synthetic **1** (*m/z* $[M+H]^+$ = 411.30).

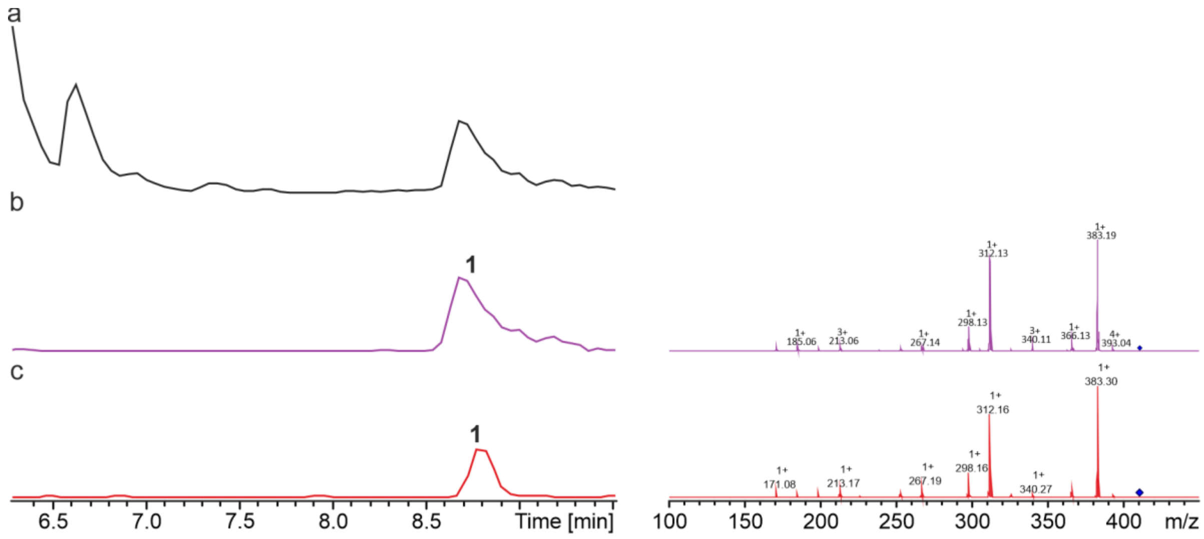

Figure S6. HPLC/MS data refers to Figure 4a (NRPS-8) of compound **1** produced in *E. coli* DH10B::*mtaA*. (a) BPC of an exemplary culture extract. (b) EIC/MS^2 of **1** (*m/z* $[M+H]^+$ = 411.30). (c) EIC/MS^2 of synthetic **1** (*m/z* $[M+H]^+$ = 411.30).

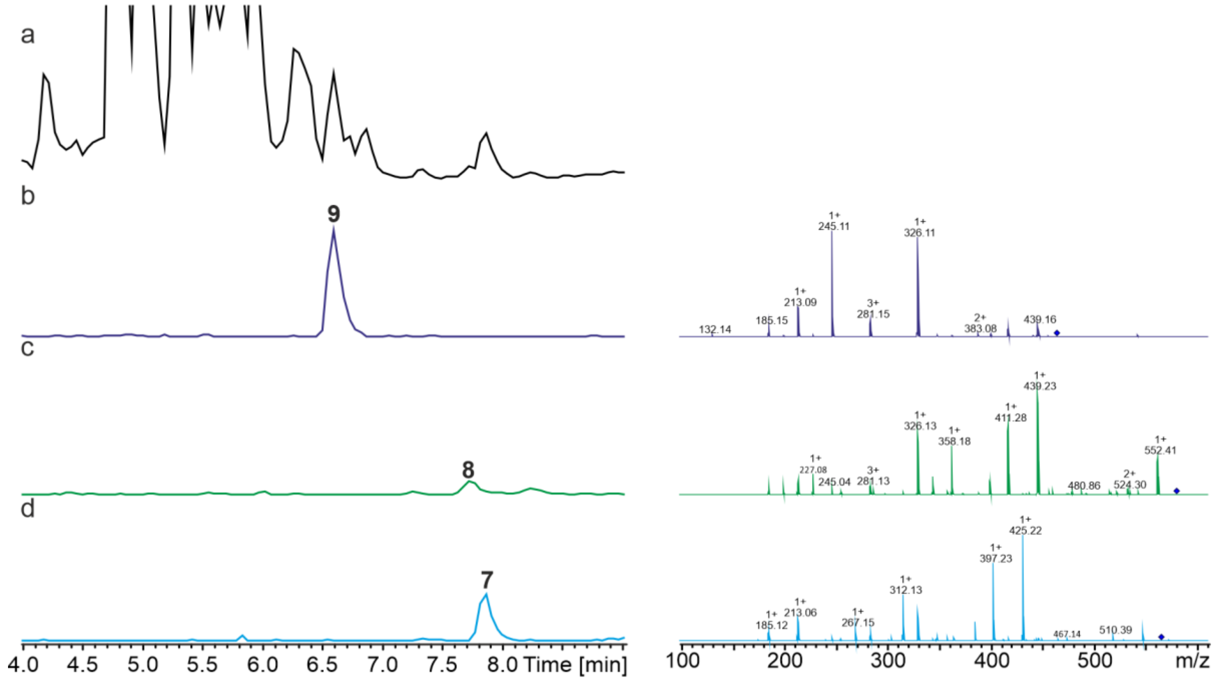

Figure S7. HPLC/MS data refers to Figure 4a (NRPS-9) of compounds **7**, **8** and **9** produced in *E. coli* DH10B::*mtaA.* (a) BPC of an exemplary culture extract. (b) EIC/MS^2 of **9** (*m/z* $[M+H]^+$ = 457.34). (c) EIC/MS^2 of **8** (*m/z* $[M+H]^+$ = 570.42). (d) EIC/MS^2 of **7** (*m/z* $[M+H]^+$ = 556.41).

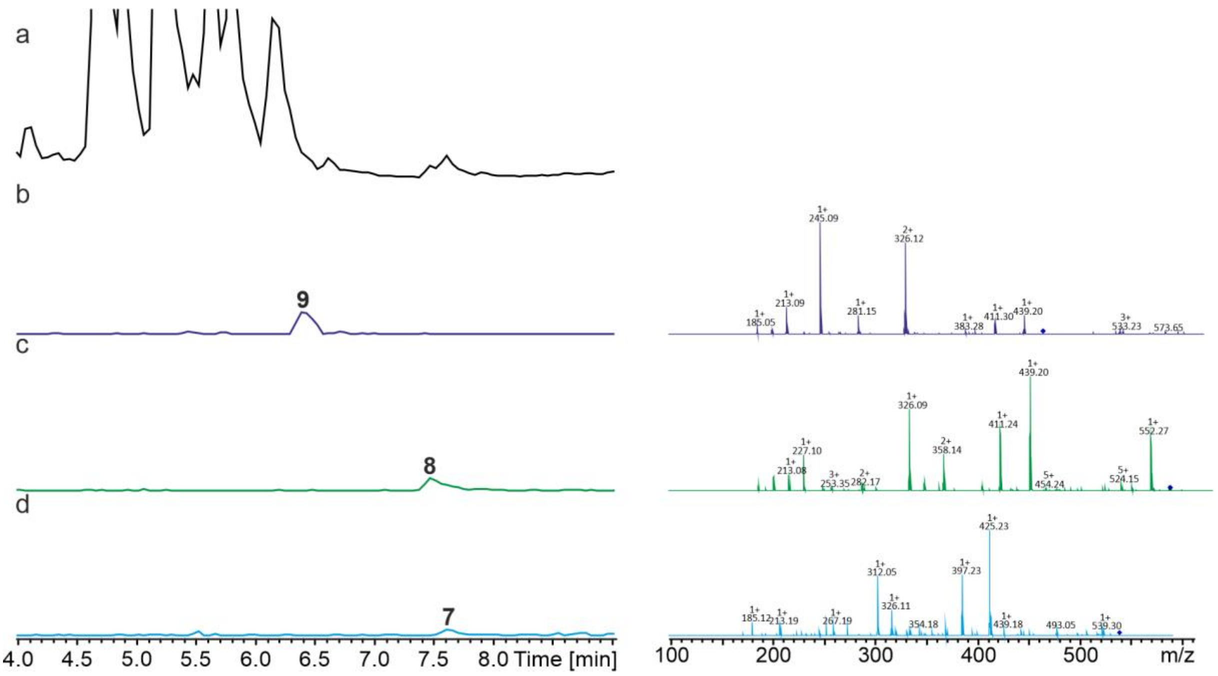

Figure S8. HPLC/MS data refers to Figure 4a (NRPS-10) of compounds **7**, **8** and **9** produced in *E. coli* DH10B::*mtaA*. (a) BPC of an exemplary culture extract. (b) EIC/MS^2 of **9** (*m/z* $[M+H]^+$ = 457.34). (c) EIC/MS^2 of **8** (*m/z* $[M+H]^+$ = 570.42). (d) EIC/MS^2 of **7** (*m/z* $[M+H]^+$ = 556.41).

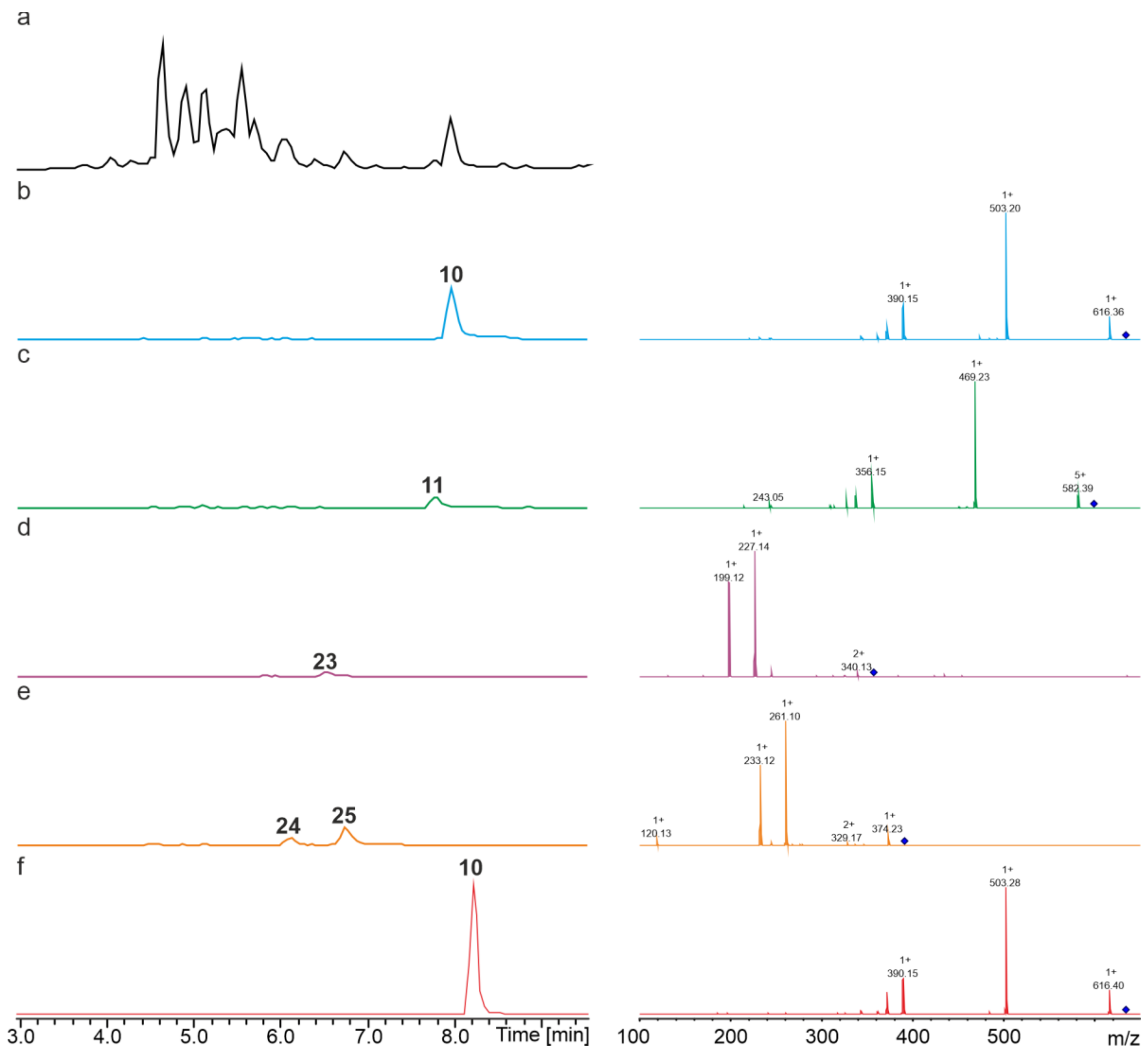

Figure S9. HPLC/MS data refers to Figure 4a (NRPS-11) of compounds **10**, **11**, **23** and **24/25** produced in *E. coli* DH10B::*mtaA*. (a) BPC of an exemplary culture extract. (b) EIC/MS^2 data of **10** (*m/z* $[M+H]^+$ = 634.38). (c) EIC/MS^2 data of **11** (*m/z* $[M+H]^+$ = 600.40). (d) EIC/MS^2 data of **23** (*m/z* $[M+H]^+$ = 358.27). (e) EIC/MS^2 data of **24/25** (*m/z* $[M+H]^+$ = 392.25). (f) EIC/MS^2 data of synthetic **10** (*m/z* $[M+H]^+$ = 634.38).

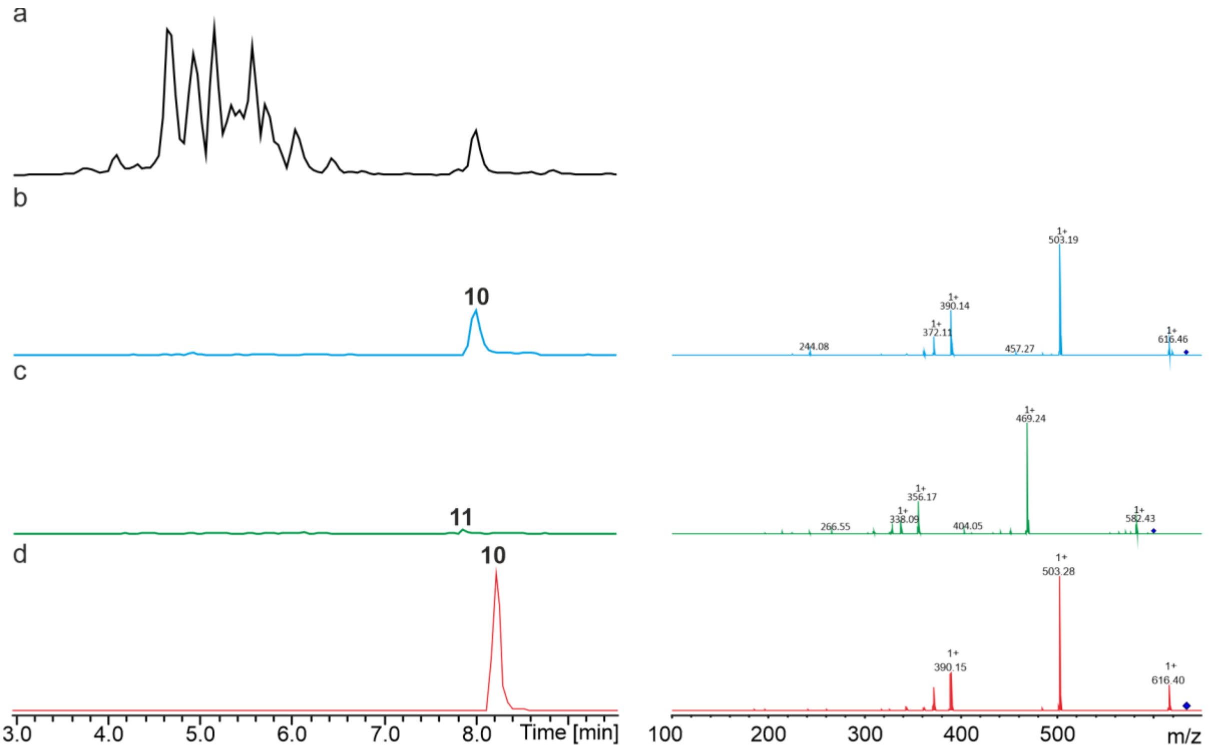

Figure S10. HPLC/MS data refers to Figure 4a (NRPS-12) of compounds **10** and **11** produced in *E. coli* DH10B::*mtaA*. (a) BPC of an exemplary culture extract. (b) EIC/MS^2 data of **10** (*m/z* $[M+H]^+$ = 634.38). (c) EIC/MS^2 data of **11** (*m/z* $[M+H]^+$ = 600.40). (d) EIC/MS^2 data of synthetic **10** (*m/z* $[M+H]^+$ = 634.38).

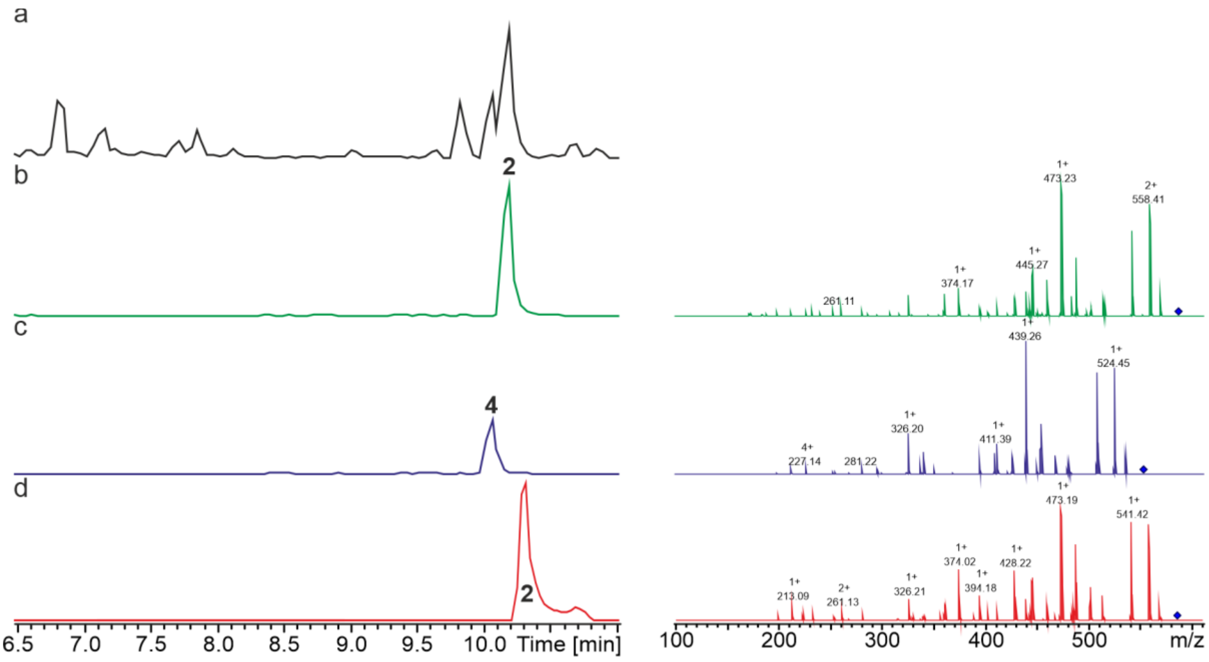

Figure S11. HPLC/MS data refers to Figure 4b (NRPS-15) of compounds **2** and **4** produced in *E. coli* DH10B::*mtaA*. (a) BPC of an exemplary culture extract. (b) EIC/MS^2 of **2** (*m/z* $[M+H]^+$ = 586.40). (c) EIC/MS^2 of **4** (*m/z* $[M+H]^+$ = 552.41). (d) EIC/MS^2 of synthetic **2** (*m/z* $[M+H]^+$ = 586.40).

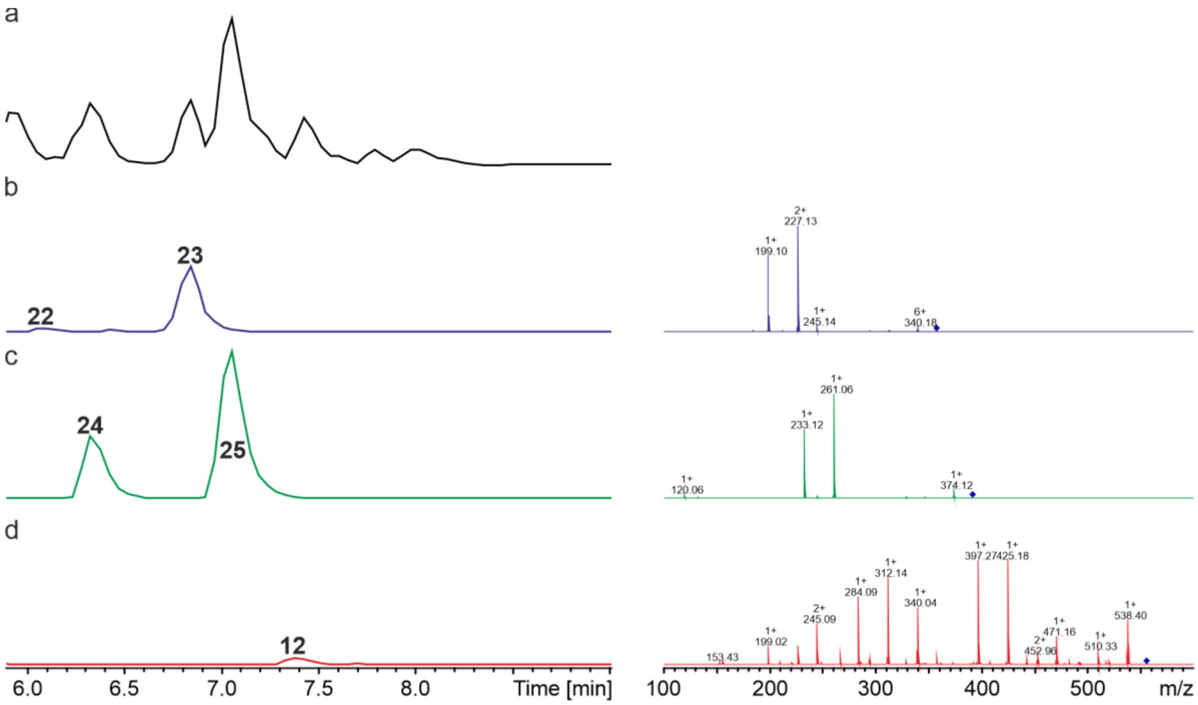

Figure S12. HPLC/MS data refers to Figure 4b (NRPS-16) of compounds **22/23**, **24/25** and **12** produced in *E. coli* DH10B::*mtaA*. (a) BPC of an exemplary culture extract. (b) EIC/MS^2 data of **22/23** (*m/z* $[M+H]^+$ = 358.27). (c) EIC/MS^2 data of **24/25** (*m/z* $[M+H]^+$ = 392.25). (d) EIC/MS^2 of **12** (*m/z* $[M+H]^+$ = 556.35).

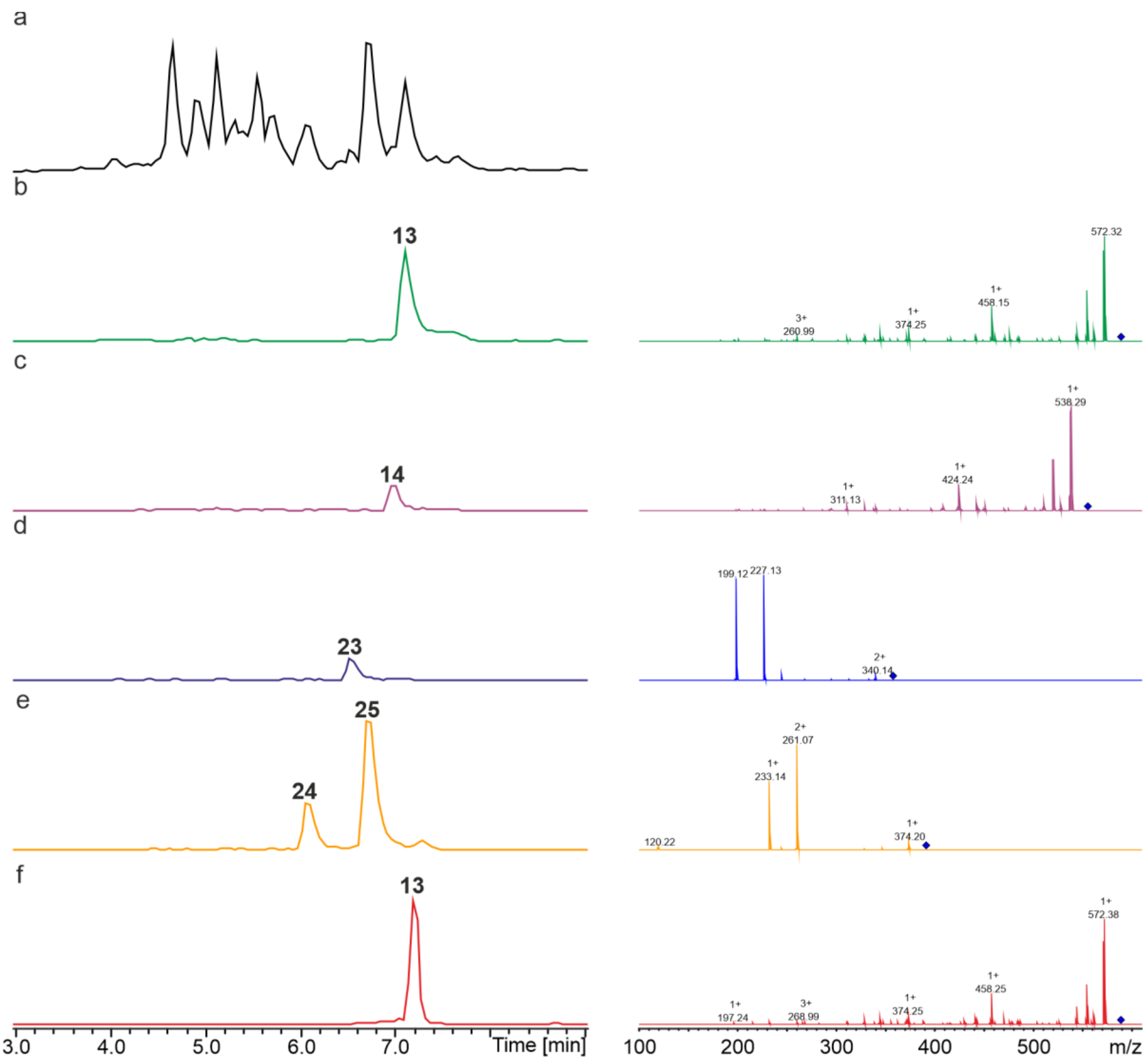

Figure S13. HPLC/MS data refers to Figure 4b (NRPS-17) of compounds **13**, **14**, **23** and **24/25** produced in *E. coli* DH10B::*mtaA*. (a) BPC of an exemplary culture extract. (b) EIC/MS^2 data of **13** (*m/z* $[M+H]^+$ = 589.33). (c) EIC/MS^2 data of **14** (*m/z* $[M+H]^+$ = 555.35). (d) EIC/MS^2 data of **23** (*m/z* $[M+H]^+$ = 358.27). (e) EIC/MS^2 data of **24/25** (*m/z* $[M+H]^+$ = 392.25). (f) EIC/MS^2 data of synthetic **13** (*m/z* $[M+H]^+$ = 589.33).

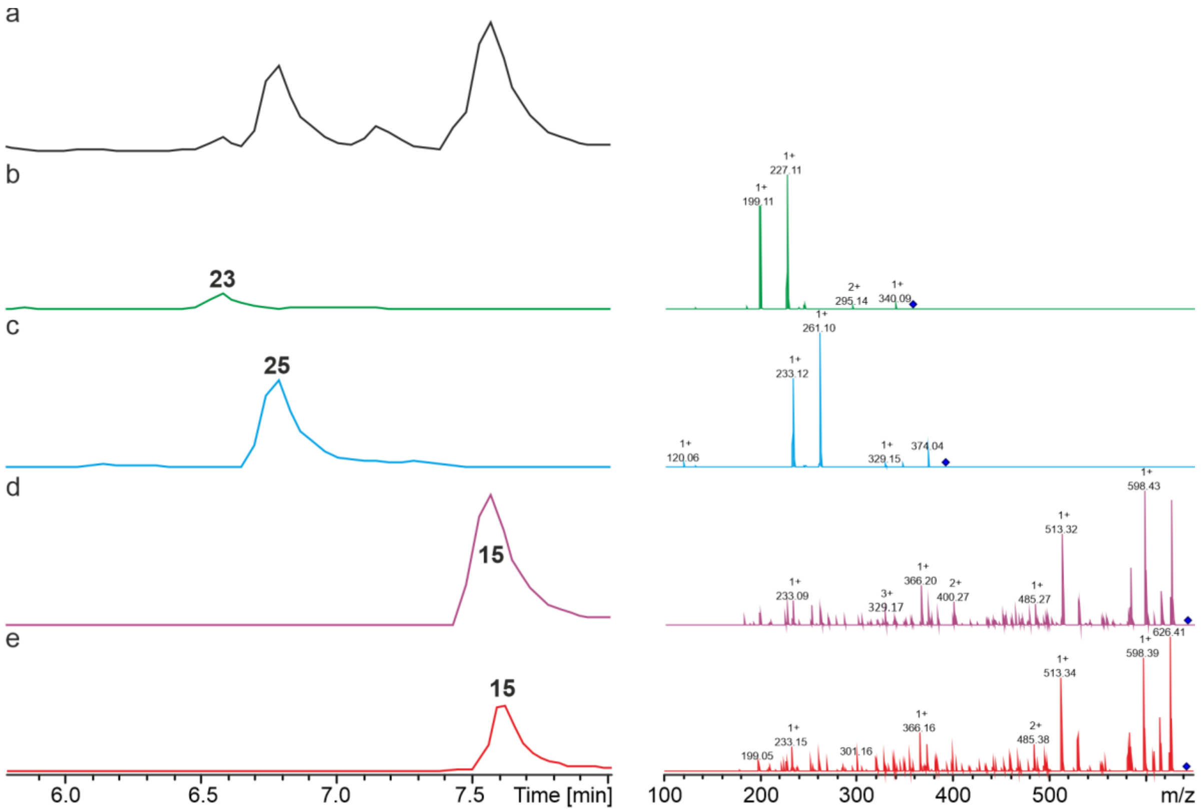

Figure S14. HPLC/MS data refers to Figure 4b (NRPS-18) of compounds **23**, **25** and **15** produced in *E. coli* DH10B::*mtaA*. (a) BPC of an exemplary culture extract. (b) EIC/MS^2 data of **23** (*m/z* $[M+H]^+$ = 358.27). (c) EIC/MS^2 data of **25** (*m/z* $[M+H]^+$ = 392.25). (d) EIC/MS^2 data of **15** (*m/z* $[M+H]^+$ = 643.43). (e) EIC/MS^2 data of synthetic **15** (*m/z* $[M+H]^+$ = 643.43).

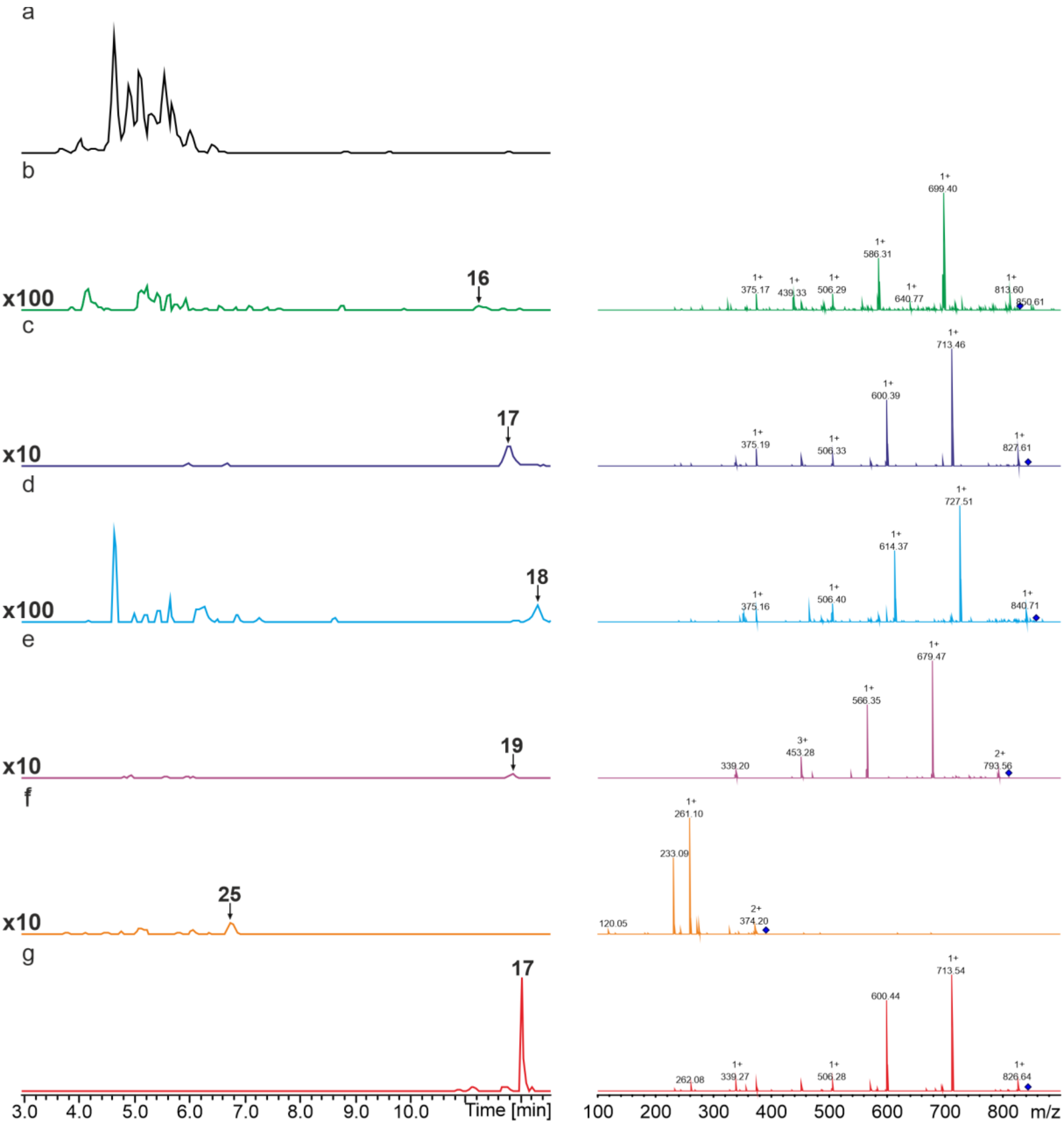

Figure S15. HPLC/MS data refers to Figure 4b (NRPS-19) of compounds **16**–**19** and **25** produced in *E. coli* DH10B::*mtaA*. (a) BPC of an exemplary culture extract. (b) EIC/MS^2 data of **16** (*m/z* [M+H]$^+$ = 830.54). (c) EIC/MS^2 data of **17** (*m/z* [M+H]$^+$ = 844.55). (d) EIC/MS^2 data of **18** (*m/z* [M+H]$^+$ = 858.57). (e) EIC/MS^2 data of **19** (*m/z* [M+H]$^+$ = 810.57). (f) EIC/MS^2 data of **25** (*m/z* [M+H]$^+$ = 392.25). (g) EIC/MS^2 data of synthetic **17** (*m/z* [M+H]$^+$ = 844.55).

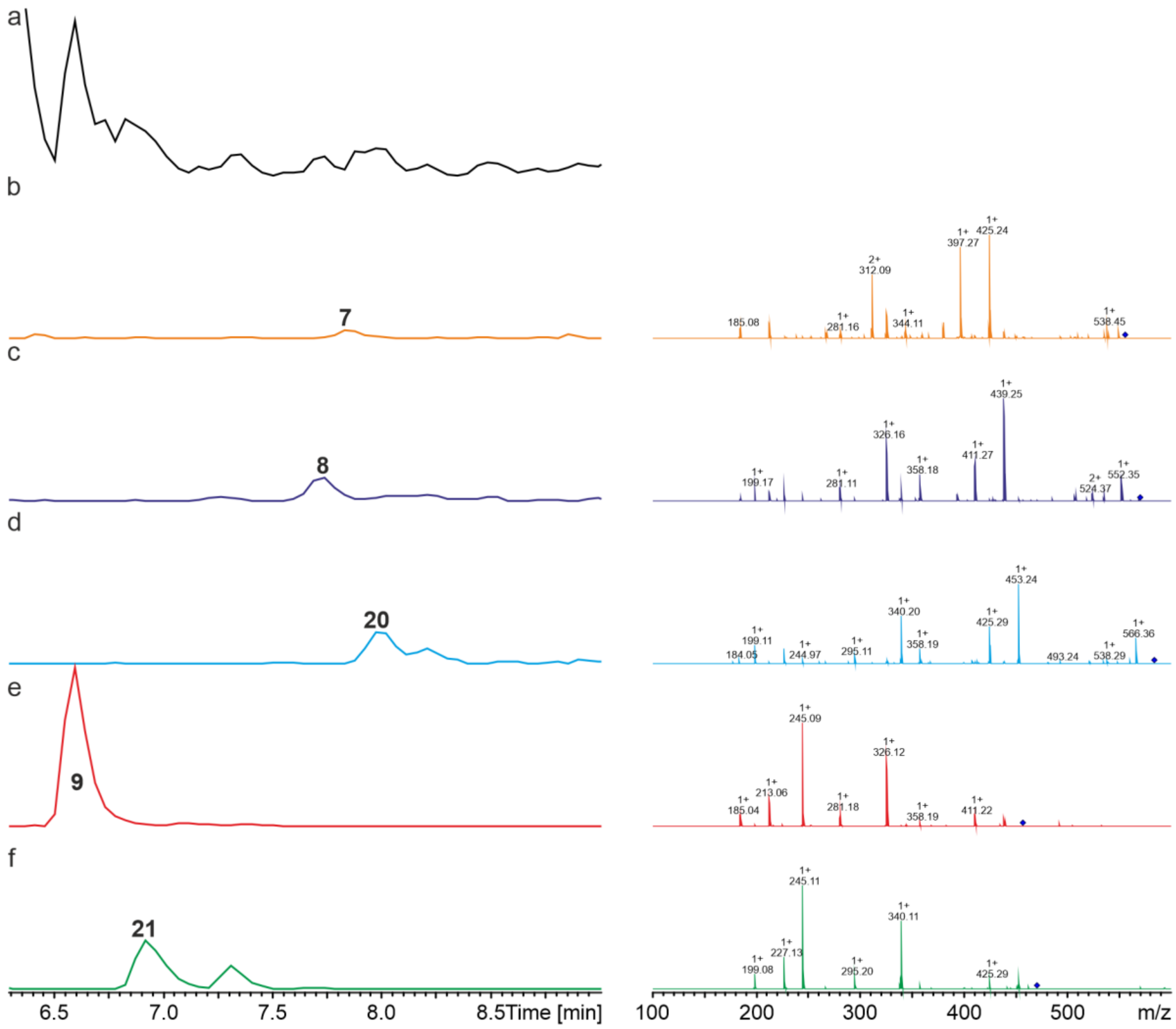

Figure S16. HPLC/MS data refers to Figure 4c (NRPS-20) of compounds **7**, **8**, **20**, **9** and **21** produced in *E. coli* DH10B::*mtaA*. (a) BPC of an exemplary culture extract. (b) EIC/MS^2 of **7** (*m/z* $[M+H]^+$ = 556.41). (c) EIC/MS^2 of **8** (*m/z* $[M+H]^+$ = 570.42). (d) EIC/MS^2 of **20** (*m/z* $[M+H]^+$ = 584.44). (e) EIC/MS^2 of **9** (*m/z* $[M+H]^+$ = 457.34). (f) EIC/MS^2 of **21** (*m/z* $[M+H]^+$ = 471.35).

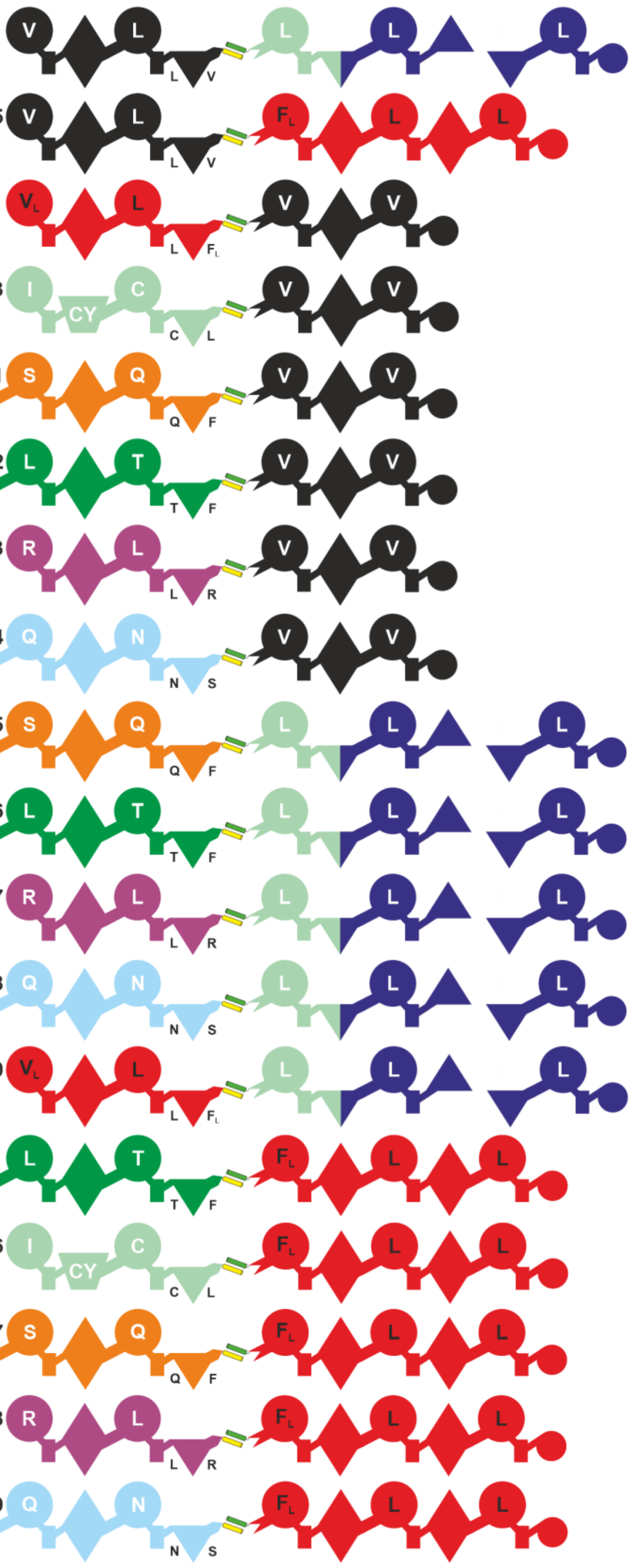

	Peptide	Production (mg l^{-1})
NRPS-9	7<br>8<br>9	2.7 ± 0.7<br>1.2 ± 0.3<br>1.3 ± 0.2
NRPS-15	2<br>3<br>4<br>5	9.3 ± 0.6<br>-<br>4.3 ± 0.2<br>-
NRPS-7	1	48.3 ± 2.8
NRPS-13	no production	
NRPS-21	no production	
NRPS-22	no production	
NRPS-23	no production	
NRPS-24	no production	
NRPS-25	no production	
NRPS-26	no production	
NRPS-27	no production	
NRPS-28	no production	
NRPS-20	7<br>8<br>9<br>20<br>21	0.5 ± 0.1<br>0.2 ± 0.02<br>0.8 ± 0.1<br>0.7 ± 0.1<br>0.3 ± 0.03
NRPS-11	10<br>11	23.8 ± 2.6<br>4.1 ± 1.1
NRPS-16	12	53.5 ± 5.0
NRPS-17	13<br>14	218.6 ± 21.3<br>46.2 ± 3.3
NRPS-18	15	39.9 ± 1.4
NRPS-19	16<br>17<br>18<br>19	0.1 ± 0.03<br>5.5 ± 0.2<br>0.4 ± 0.04<br>0.9 ± 0.3

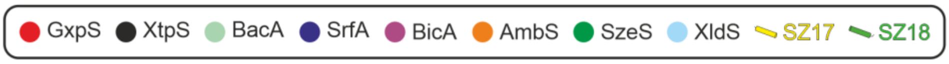

Figure S17. A schematic representation of all bipartite type S NRPSs (NRPS-7, -9, -11, -13, -15 – -28) using subunit 1 building blocks from GxpS, XtpS, BacA, AmbS XldS, SzeS and BicA combined with subunit 2 building blocks from GxpS, XtpS and RtpS. All constructed subunits 1 and 2 with attached synthetic zippers were functional, as at least one functional combination could be observed in each case. Co-expression of two subunits each led to detectable peptide amounts in 9 out of 18 cases. Non-productive type S NRPS combinations involved subunits 2, either from XtpS (type S NRPSs: 7, 13, 21, 22, 23, and 24) or RtpS (type S NRPSs: 25 – 28). From these non-producing type S NRPSs it can be deduced that: (I) the TE domain from XtpS has a very narrow substrate range, at least when it comes to positions 1 and 2 of the synthesised peptides; and (II) that subunits of Gram-positive and -negative origin can be functionally combined *in trans*, if the additive negative effect of introduced impairments is not too great, i.e. the substrate specificity of involved TE and C domains as well as the formed chimeric C-A interface. In conclusion, especially for subunits of only distantly related bacteria it is imperative to keep these caveats in mind.

a

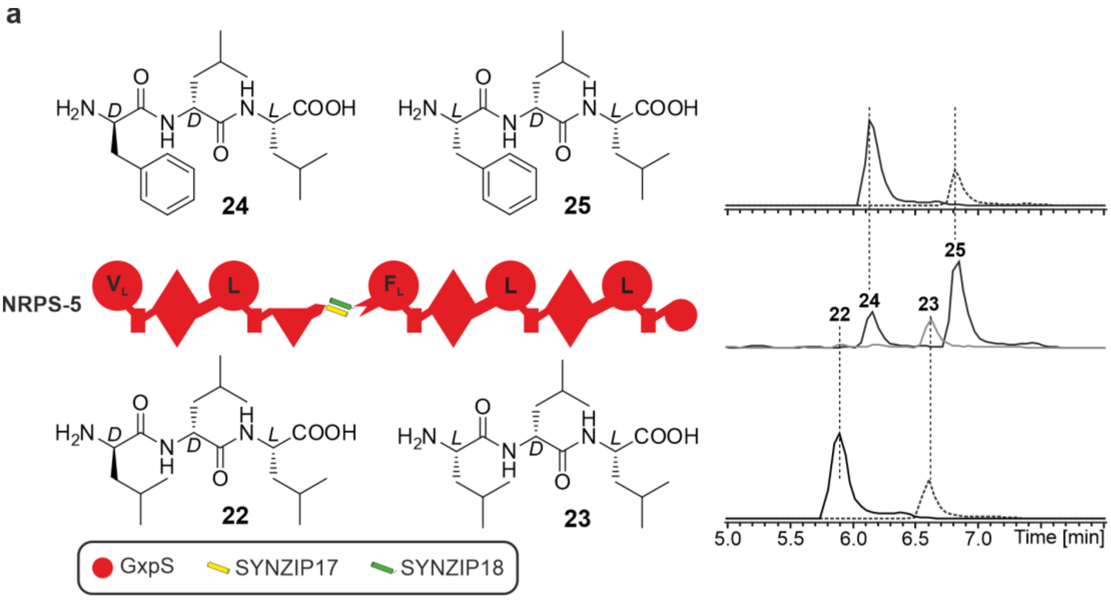

b

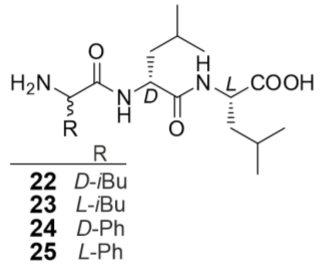

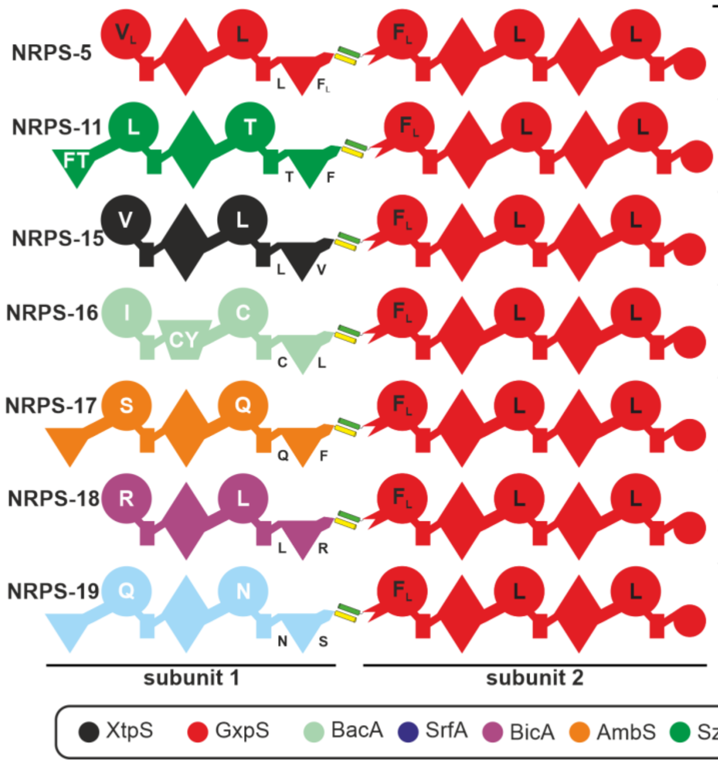

	Peptide	Production (mg l[-1])
NRPS-5	22	-
	23	3.6±0.1
	24	5.3±0.1
	25	16.1±0.2
NRPS-11	22	0.1±0.01
	23	1.4±0.2
	24	2.0±0.3
	25	5.6±1.2
NRPS-15	22	0.1±0.01
	23	0.7±0.1
	24	1.4±0.1
	25	4.9±0.5
NRPS-16	22	1.1±0.3
	23	20.0±1.2
	24	18.3±0.5
	25	47.5±2.1
NRPS-17	22	0.2±0.1
	23	5.1±0.6
	24	12.9±0.7
	25	37.7±2.4
NRPS-18	22	0.2±0.1
	23	10.2±0.6
	24	2.3±0.04
	25	86.2±2.8
NRPS-19	22	-
	23	-
	24	0.2±0.02
	25	0.4±0.03

Figure S18. (a) Production of *D*/*L*-tripeptides exemplary of NRPS-5 (Fig. 3). The tripeptide production is related to the unpaired activity of GxpS subunit 2 resulted in the production of peptides **22/23** and **24/25**. The different epimers could be identified by their retention times. (b) Tripeptide **22/23** and **24/25** amounts and yields (determined in triplicates (n=3)) are given for all NRPS systems shown in Fig. 3. The colour code of the NRPS subunits is depicted at the bottom of the figures. The domain assignment is as described in Fig. 3 and 4.

4 References

[1] C. Fu, W. P. Donovan, O. Shikapwashya-Hasser, X. Ye, R. H. Cole, *PLoS One* **2014**, *9*, e115318.
[2] K. E. Thompson, C. J. Bashor, W. A. Lim, A. E. Keating, *ACS Synth. Biol.* **2012**, *1*, 118–129.
[3] K. A. J. Bozhüyük, F. Fleischhacker, A. Linck, F. Wesche, A. Tietze, C.-P. Niesert, H. B. Bode, *Nat. Chem.* **2018**, *10*, 275–281.
[4] K. A. J. Bozhüyük, A. Linck, A. Tietze, J. Kranz, F. Wesche, S. Nowak, F. Fleischhacker, Y.-N. Shi, P. Grün, H. B. Bode, *Nat. Chem.* **2019**, *11*, 653–661.
[5] C. Kegler, F. I. Nollmann, T. Ahrendt, F. Fleischhacker, E. Bode, H. B. Bode, *ChemBioChem* **2014**, *15*, 826–828.
[6] H. B. Bode, D. Reimer, S. W. Fuchs, F. Kirchner, C. Dauth, C. Kegler, W. Lorenzen, A. O. Brachmann, P. Grün, *Chemistry* **2012**, *18*, 2342–2348.
[7] D. Hanahan, *J. Mol. Biol.* **1983**, *166*, 557–580.
[8] O. Schimming, F. Fleischhacker, F. I. Nollmann, H. B. Bode, *ChemBioChem* **2014**, *15*, 1290–1294.
[9] S. W. Fuchs, C. C. Sachs, C. Kegler, F. I. Nollmann, M. Karas, H. B. Bode, *Anal. Chem.* **2012**, *84*, 6948–6955.
[10] E. Bode, A. K. Heinrich, M. Hirschmann, D. Abebew, Y.-N. Shi, T. D. Vo, F. Wesche, Y.-M. Shi, P. Grün, S. Simonyi et al., *Angew. Chem., Int. Ed.* **2019**, *58*, 18957–18963.
[11] D. Konz, A. Klens, K. Schörgendorfer, M. A. Marahiel, *Chem. Biol.* **1997**, *4*, 927–937.
[12] P. Cosmina, F. Rodriguez, F. de Ferra, G. Grandi, M. Perego, G. Venema, D. van Sinderen, *Mol. Microbiol.* **1993**, *8*, 821–831.
[13] W. Lorenzen, T. Ahrendt, K. A. J. Bozhüyük, H. B. Bode, *Nat. Chem. Biol.* **2014**, *10*, 425–427.
[14] C. Kegler, H. B. Bode, *Angew. Chem., Int. Ed.* **2020**, *59*, 13463–13467.
